# All‐in‐One, Wireless, Multi‐Sensor Integrated Athlete Health Monitor for Real‐Time Continuous Detection of Dehydration and Physiological Stress

**DOI:** 10.1002/advs.202403238

**Published:** 2024-07-01

**Authors:** Ka Ram Kim, Tae Woog Kang, Hodam Kim, Yoon Jae Lee, Sung Hoon Lee, Hoon Yi, Hyeon Seok Kim, Hojoong Kim, Jihee Min, Jud Ready, Melinda Millard‐Stafford, Woon‐Hong Yeo

**Affiliations:** ^1^ George W. Woodruff School of Mechanical Engineering Georgia Institute of Technology Atlanta GA 30332 USA; ^2^ Wearable Intelligent Systems and Healthcare Center (WISH Center) Institute for Matter and Systems Georgia Institute of Technology Atlanta GA 30332 USA; ^3^ School of Electrical and Computer Engineering Georgia Institute of Technology Atlanta GA 30332 USA; ^4^ Department of Biology College of Arts and Sciences Emory University Atlanta GA 30322 USA; ^5^ Electro‐Optical Systems Laboratory Georgia Tech Research Institute Atlanta GA 30332 USA; ^6^ School of Biological Sciences Georgia Institute of Technology Atlanta GA 30332 USA; ^7^ Wallace H. Coulter Department of Biomedical Engineering Georgia Tech and Emory University School of Medicine Atlanta GA 30332 USA; ^8^ Parker H. Petit Institute for Bioengineering and Biosciences Institute for Robotics and Intelligent Machines Georgia Institute of Technology Atlanta GA 30332 USA

**Keywords:** all‐in‐one wearable, athlete health monitor, chest patch, dehydration detection, physiological stress, smart mouthguard

## Abstract

Athletes are at high risk of dehydration, fatigue, and cardiac disorders due to extreme performance in often harsh environments. Despite advancements in sports training protocols, there is an urgent need for a non‐invasive system capable of comprehensive health monitoring. Although a few existing wearables measure athlete's performance, they are limited by a single function, rigidity, bulkiness, and required straps and adhesives. Here, an all‐in‐one, multi‐sensor integrated wearable system utilizing a set of nanomembrane soft sensors and electronics, enabling wireless, real‐time, continuous monitoring of saliva osmolality, skin temperature, and heart functions is introduced. This system, using a soft patch and a sensor‐integrated mouthguard, provides comprehensive monitoring of an athlete's hydration and physiological stress levels. A validation study in detecting real‐time physiological levels shows the device's performance in capturing moments (400–500 s) of synchronized acute elevation in dehydration (350%) and physiological strain (175%) during field training sessions. Demonstration with a few human subjects highlights the system's capability to detect early signs of health abnormality, thus improving the healthcare of sports athletes.

## Graphical abstract

This article introduces an all‐in‐one, multi‐sensor integrated wearable system, enabling wireless, real‐time, continuous monitoring of saliva osmolality, skin temperature, and heart functions.

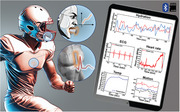

## 1 Introduction

Recent advancements in wearable electronics for health monitoring have significantly expanded their application spectrum.^[^ ^1^, ^2^, ^3^ ^]^ These devices now integrate multifaceted monitoring parameters, encompassing sensor signal metrics (such as force, accelerometry, gyroscopic, and temperature),^[^ ^4^, ^5^, ^6^, ^7^ ^]^ electrophysiological signals (including electromyography, electrocardiography, and electrodermal activity),^[^ ^8^, ^9^, ^10^, ^11^, ^12^, ^13^ ^]^ and biomarkers^[^ ^11^, ^14^, ^15^, ^16^ ^]^ (like metabolite levels, hydration status, and various molecules from blood, urine, saliva, and tears). Such comprehensive data collection enables the identification of critical health indicators, fosters the discovery of novel correlations among diverse biomarkers, and aids in the proactive identification of potential health risks.^[^ ^17^, ^18^, ^19^, ^20^ ^]^ The incorporation of multimodal monitoring in wearable devices offers significant advantages, particularly in the realm of medical diagnostics. These devices can pinpoint specific health abnormalities by analyzing trends across multiple metrics, providing valuable insights that inform medical decision‐making. However, integrating multimodal monitoring poses distinct challenges, particularly regarding device placement for optimal data acquisition. For instance, electrophysiological monitoring necessitates strategic placement near the chest or specific muscle groups. Similarly, physiological sensors need to be positioned in alignment with the source organ, such as using ocular sensors for tear analysis and oral sensors for saliva analysis.^[^ ^21^, ^22^, ^23^, ^24^ ^]^ In the context of user experience, there is an increasing demand for wireless, non‐invasive wearable devices. The trend toward minimizing physical obstructions and discomfort necessitates designs that reduce the number of devices attached to the body, even in wireless configurations.^[^ ^25^ ^]^ Accordingly, our research focuses on the assessment of dehydration in athletes through continuous monitoring of saliva and electrophysiological parameters. Athletic wearables should be robust and capable of withstanding sweating from intense physical activity, environmental factors like heat and humidity, and potential impairment from salts and other metabolites.^[^ ^26^ ^]^ Common practices have emerged to address the requirement, such as patch‐type devices utilizing adhesive tape rather than constrictive banding and encapsulating with appropriate modulus elastomer. Superior adhesion property between skin and substrate enables conformal contact that offers enhanced signal sampling accuracy and sensitivity.^[^ ^27^, ^28^ ^]^ The development of ultra‐thin electrodes and fabric‐based sensors integrated into smart clothing also represents significant advancements in this domain. Athletes present a unique subset of subjects for health monitoring research.^[^ ^29^ ^]^ Despite their generally excellent physical health, the intense nature of their physical activities exposes them to risks such as physical trauma, cardiac abnormalities, and significant fluid loss. Among the risks, dehydration can cause cognitive performance impairment and severe complications related to muscle and heart conditions from imbalanced ion profiles in the body.^[^ ^26^, ^30^, ^31^ ^]^ The accurate assessment of dehydration and heat strain in athletes is complex, with potential indicators including urine and blood analysis, thirst perception, and various cardiac monitoring markers like heart rate (HR), blood pressure, and heart rate variability (HRV).^[^ ^32^ ^]^ The most practical analyses are based on the endpoint assay of urine analysis and body mass measurement at pre‐ and post‐exercise, which is not feasible for wearable settings.^[^ ^33^, ^34^, ^35^ ^]^ As indirectly applicable markers, saliva osmolality shows specificity with moderate sensitivity to dehydration assessment.^[^ ^32^, ^36^, ^37^ ^]^ Yet, salivary measures are considered immature biomarkers due to confounders influencing validity. Thus, while each of these markers has been extensively studied, and numerous protocols have been developed, a gap still needs to be seen between the clinical standards required for accurate assessment and the practical functionality of wearable devices in sports contexts.^[^ ^38^ ^]^ This disparity underscores the critical need for translational research between the ideal design and the actual performance of wearable health monitoring technologies in athletic settings. In this context, we present a combinatory approach to monitor continuous saliva and cardiac monitoring markers during physical exercise, utilizing a smart mouthguard and soft chest patch designed to track saliva osmolality, electrocardiogram (ECG), and heart rate derivatives. Paired wearable devices could provide continuous monitoring features of saliva osmolality and physiological strain to assess the risk of heat‐related illness exacerbated by dehydration. This method offers a practical solution for real‐time, non‐invasive assessment of hydration status, highlighting its potential in health monitoring applications.

## 2 Result and Discussion

### 2.1 Overview of an All‐in‐One, Multi‐Sensor Integrated Athlete Health Monitoring System

**Figure 1:**
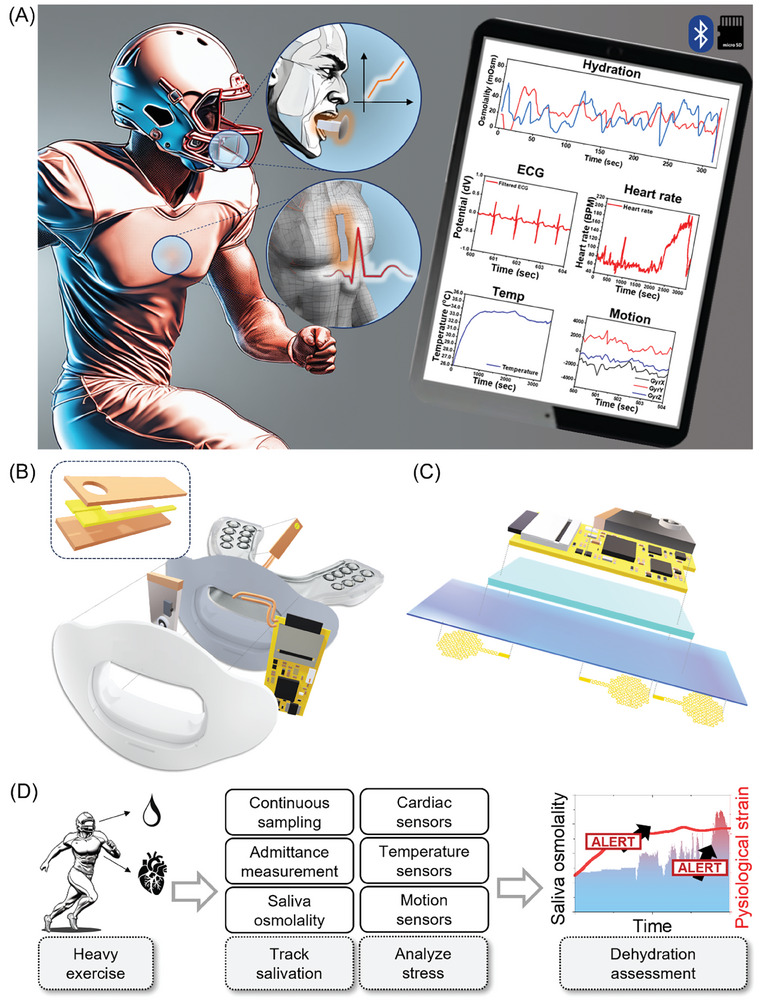
Overview of an all‐in‐one wearable system for athlete health monitoring. A) Illustration depicting a wearable smart mouthguard and chest patch for continuous dehydration assessment and health monitoring through saliva and cardiovascular signal analysis. B) Detailed illustration of a smart mouthguard. C) Detailed illustration of a chest‐wearable soft patch. D) Flow chart describing the aim of combinatory monitoring strategies for a dehydration warning scheme using physiological strain and saliva osmolality.

As shown in **Figure** **1**A, the goal of this system is to monitor saliva osmolality changes with changes in total body water (up to ≈2% body mass loss) associated with cardiovascular changes during exercise training. To facilitate these features, a pair of mouthguards and chest patches are utilized as a substrate for the thin, flexible, humidity‐durable wearable electronics capable of providing a conformal and minimum irritating experience to the users. Figure 1B illustrates a smart mouthguard featuring a flexible electrical circuit integrated into the lip guard. This mouthguard is equipped with a micro‐gap electrode specifically designed for saliva analysis, enabling it to monitor osmolality (oral solute concentration) as part of its health monitoring capabilities.^[^ ^39^ ^]^ To do so, the impedance measurement‐based osmolality analysis is applied through the impedance‐converting component (Figure S1A, Supporting Information). A micro‐gap sensor tip for measuring saliva osmolality is placed on the side of the tooth chewing pad to ensure the continuous supply of saliva from the tongue. As another pair of proposed health monitoring system, Figure 1C shows the schematical composition of the soft chest patch. It is designed to attach to the upper sternum to collect electrocardiography, skin temperature, and motion‐related parameters, such as gyroscope and accelerometer (Figure S1B, Supporting Information). Patches are designed to attach to the rigid parts of the human chest to register the cardiac muscle‐generated electrophysiological potentials. Furthermore, devices have been encapsulated with elastomer layers to ensure the actual training action's humidity‐ and impact endurance. In the present study, we introduce a wearable system to support the methodology for assessing dehydration, utilizing changes in saliva content and HR, ECG, and HRV indicators. This led us to integrate two distinct physiological parameters—those from the oral cavity and heart—to monitor and infer the status of mild dehydration in individuals engaged in intense physical exercise. As illustrated in Figure 1D, water content loss in heavy exercise will cause several physiological changes with elevated HR response above that compared to exercise when dehydration is prevented, which is difficult to track in real‐time using body mass change or monitoring fluid such as blood, urine, and saliva.^[^ ^35^, ^40^ ^]^ Among them, our system collects signals from the heart and saliva. This is achieved through the continuous tracking of both saliva composition and cardiac physiological signals, providing a more comprehensive and non‐invasive approach to monitor athlete safety. In the case of saliva, continuous sampling and measurement of admittance can estimate the saliva osmolality as a progressive rise would be consistent with a loss of body fluid. Soft electrodes with flexible and stretchable features can also address fine ECG readings. ECG reveals the electrical activity of the heart along with HR and HRV, which can collectively track cardiovascular strain, especially when combined with skin temperatures. Combining these features, we aimed to determine the potential applicability of our paired wearable devices in exercise eliciting mild‐moderate dehydration. In this regard, the results of the evaluation from the laboratory and the field are studied, with notable moments in exercise monitoring discussed.

### 2.2 Design, Structure, and Fabrication of the Smart Mouthguard and Soft Chest Patches

**Figure 2:**
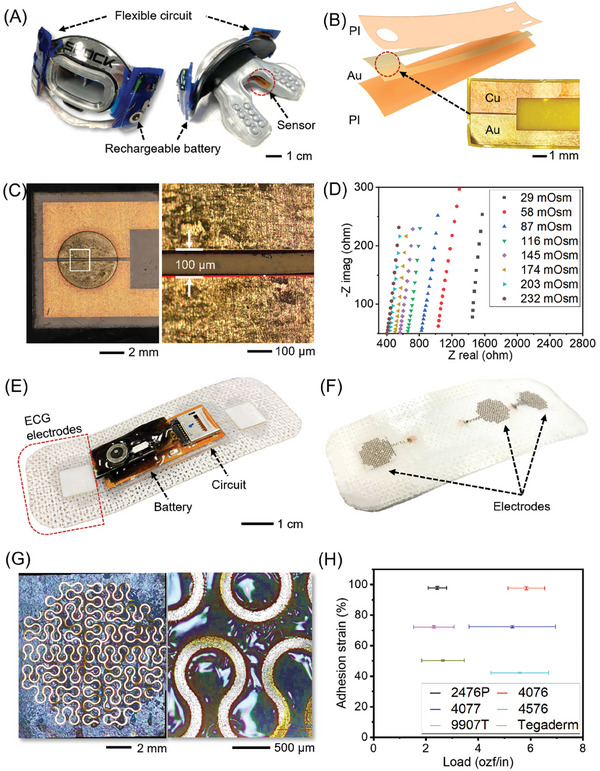
Fabrication and characterization of smart mouthguards and soft chest patches. A) Front and backside view of a mouthguard with integrated, flexible PCB and rechargeable battery on lip guard, micro‐gap electrode on the inner side wall. B) Fabrication of an electrode using a heat‐responsive adherent polyimide film to cover a conductive foil layer. C) Microscopic images of micro‐gap electrodes showing a circular gold surface with 100 µm gap. D) Electrical impedance spectroscopy data from the electrode in standard calibrator, demonstrating linear impedance proportional to osmolar levels. E) Front and backside view of a soft chest patch. F) Fabricated nanomembrane electrodes on the adherent substrate. G) Microscopic images of the serpentine gold electrode. H) Peeling test results illustrating adhesion strain according to different substrates and loading.

A smart mouthguard consists of a soft and flexible micro‐gap electrode integrated into the flexible electrical devices capable of detecting saliva osmolality on the commercial mouthguard with encapsulations. **Figure** **2**A shows a photo of the device's front and back sides that comprise a flexible printed circuit board (PCB) circuit, rechargeable lithium‐ion polymer (Li‐po) battery (Adafruit, USA) on the lip guard, and saliva osmolality sensing tip connected with enameled copper wires from flexible PCB circuit. As a saliva osmolality sensing tip, a flexible micro‐gap electrode is attached to the side wall of the mouthpiece and is designed to collect a continuous saliva supply from the salivary gland during the constant swallowing action. Precisely, the position of the electrode on the inner side of the mouthpiece is targeted to collect saliva from the parotid gland,^[^ ^41^ ^]^ which can reflect plasma levels indicative of hydration status. Subsequently, involuntary movement of the tongue can deliver and wet the micro‐gap electrode. The sensor tip position can be changed to the requirements of the user experience and collect the successful flow from the salivary gland. The sensing tip is fabricated from the simple heat press of the heat‐responsive polyimide (PI) film with the micro‐patterned gold‐electroplated copper foil (Figure 2B). The patterned micro gap was designed to maintain the 100‐micron distance, and Figure 2C shows a fine gap between the patterned electrodes. Since the PI and metal foil layer would not be stretchable yet flexible, firm adhesion for the curved polymer surface of the mouthpiece is successful. Figure 2D shows the serial response of the impedance that matches the increment of the standard osmolality sample concentration. Electrical impedance spectroscopy (Gamry, Germany) results from micro‐gap electrodes, reflecting that the impedance has been shown to be proportional to the various concentrations of samples. As a test, the concentration range has been set to cover the expected human saliva osmolality ranges (25–250 mOsm).^[^ ^42^ ^]^ The ohmic resistance (Z‐Real), the intercept on the real impedance axis, represents the characteristics of ionic conductivity of the electrolyte as electrolyte resistance. Each intercept resistance value, which can be converted into the admittance values, is inversely proportional to the standard osmolar testing sample concentration. As for the soft chest patches, multiple sensory circuits are integrated to track ECG, temperature, and motions from the user. Each target is selected as a critical health parameter for athletic performance and health condition monitoring. The size of the device (18 × 46 mm) can be attached to the upper sternum to ensure minimized strain disturbance in torso activity and collect the electrophysiological signals from the heart more closely than other parts of the body. The device is consistent with a series of layers of electrode‐integrated medical tape, flexible PCB, and rechargeable Li‐po battery with a custom mix of silicone‐based elastomeric encapsulation for the electrical components to protect from humidity, foreign substances, and physical impact (Figure 2E). In order to capture the potential for ECG, flexible and ultrathin electrodes are applied.^[^ ^43^, ^44^ ^]^ The electrodes were serially deposited through an electron beam evaporator to fabricate a 10 nm chromium (Cr) and 100 nm gold (Au) layer on the PI film. From this nanomembrane electrode, octagonal serpentine patterning was conducted to facilitate conformal contact on the skin curvatures. After that, femtosecond laser micromachining of the PI‐Cr‐Au film is utilized, and the patterned electrodes are transferred to medical tape (Figure 2F). Additionally, on the opposite side of the serpentine electrode above the tape, a semi‐rigid plastic backing was added. This strain‐isolating design ensures stable signal acquisition by mitigating electrode deformation caused by physical activities. The combined electrode/tape/rigid‐backing configuration offers to endure different levels of mechanical strain during various movements, resulting in enhanced signal quality. As shown in Figure 2G, the gold serpentine nanomembrane electrode faces the adherent surface of the medical tape, and the nanomembrane electrodes are on the surface. Capturing high signal‐to‐noise ECG relies on the adhesion properties between soft body curved surfaces. As for the electrode's substrate, a medical tape is carefully selected, determining enough adhesion, safety, and user's comfort. As discussed in our previous work,^[^ ^45^ ^]^ the essential property of the tape is its adequate adhesion when mounted on the sweaty skin of athletes in this study. We tested five commercial medical tapes to identify the best one, as summarized in Figure 2H. Although the candidates of adhesion substrate for the flexible PCB circuits are well known with the product specification, each candidate was tested with the practical scenario of the human skin during heavy workout sessions. To ensure better signal quality through superior adhesive properties to the body surface, tests were conducted on the 180° peeling strength on dry/sweaty skin comparison (Figure S2A and B, Supporting Information). In detail, 6‐inch tapes were attached to the participant's arm and wrapped twice with a 12‐inch‐wide wrap. Light jogging was conducted for 30 min in sunny, 25 °C conditions to induce sweat. The average sweat quantity absorbed by the tapes on the participants' skin was 0.3422 g (Figure S2C, Supporting Information). The tape was then peeled using an automated force meter. All peeling tests were performed five times. The adhesion strain percentage indicates the fraction of peeling strength between dry and sweaty skin, and the load indicates the ounce force per width of the sample tapes, which can be translated as an upper right side in the graph can maintain its adhesion properties in sweaty conditions on human skin with higher strength. As a result, the 4076 medical tape is found to have the highest adhesion in sweaty conditions and utilized in further use as the substrate of the soft chest patch.

### 2.3 Validation of the Saliva Osmolality Sensor on the Smart Mouthguard

**Figure 3:**
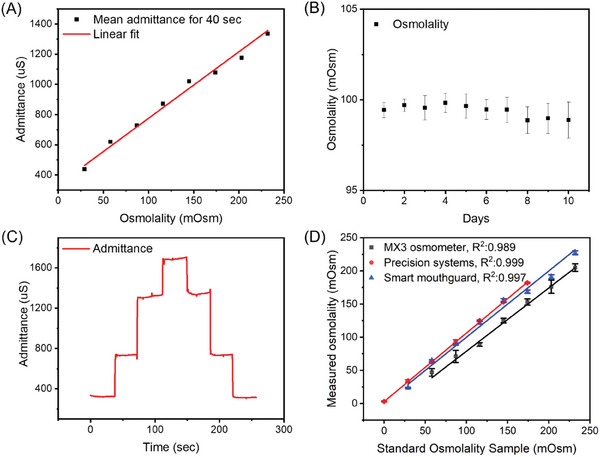
Validation of the smart mouthguard's performance. A) Device calibration with standard osmolar samples ranging from 29 to 232 mOsm. B) Results showing stable signals from the micro‐gap electrode immersed in artificial saliva at 100 mOsm. C) Demonstration of continuous osmolar change detection using 29, 87, 174, 232, 174, 87, and 29 mOsm sequentially. D) Comparison of calibration data between a commercial saliva osmometer (MX3), a gold standard precision system (Micro‐Osmette osmometer), and a smart mouthguard developed in this work.

The validation of our micro‐gap sensor tip, integrated into a smart mouthguard for saliva osmolality sensing, was meticulously conducted to calibrate the sensor and ascertain its suitability for real saliva samples. Traditional osmolality testing, which primarily relies on freezing point depression analysis, is considered the gold standard.^[^ ^46^ ^]^ In contrast, our admittance‐based osmolality test represents an indirect approach. Notably, in cases of mild to moderate dehydration, saliva osmolality variations are closely correlated with fluctuations in ion concentrations. Consequently, we have chosen and evaluated the use of admittance values in fluids, particularly focusing on the electrolytes, to gauge their sensitivity. This approach aims to provide a more direct and practical measure of monitoring saliva osmolality, especially relevant in the context of dehydration assessment on compact, robust wearable electronics. In the initial phase of our study, we evaluated the osmolality analysis using standard samples designed for a freezing point osmometer (Micro‐Osmette, Precision Inc, USA). As depicted in **Figure** **3**A, we calibrated and verified the linear proportionality of admittance across a concentration range of 25 to 230 mOsm. To clarify the osmolality range of interest, the specific range of 25 to 230 mOsm was selected based on the previous study.^[^ ^42^ ^]^ Furthermore, a commercial saliva analysis device (MX3 Diagnostics) defines hydration levels as follows: 1) hydrated: below 65 mOsm, 2) mildly dehydrated: 65–100 mOsm, 3) moderately dehydrated: 100–150 mOsm, and 4) severely dehydrated: above 150 mOsm. Admittance analysis process acknowledges that osmolar changes in the sample solution are dependent on the diffusion of solutes in the solvent, necessitating a certain duration for the admittance data collection to allow diffusion to reach equilibrium. For this calibration study, a duration of 40 s was established, with the average data from this period used to construct the calibration curve. The osmolality sensor's circuitry was designed with an impedance‐measuring component tailored for biomedical applications. Initially, the output value is in impedance (Ohm), which is then converted to admittance; as admittance is the reciprocal of impedance, our results display a linear increase correlating with higher osmolality values. It is crucial to understand that osmolality reflects the concentration of particles in a given solvent volume. Consequently, the reactivity of saliva's specific contents, including ions and both inorganic and organic components, is a significant factor affecting the quality of the calibrated signal. Therefore, ensuring chemical resistance and signal stability is important in maintaining the integrity and accuracy of our measurements. Furthermore, the protein absorption and unintended solid contents, including alkali or acidic profiles in the mouth environment, should be considered as a potential threat to the gap electrode integrity during the admittance measurement. Figure 3B displays the results of a stability test conducted to assess potential sensing deficiency in the calibrated sensor. For this test, we employed a triplicate measurement using a 100 mOsm‐spiked artificial saliva sample (provided by Pickering Laboratories, Inc., USA) over a duration of 10 days, observing a maximum measurement fluctuation of 3.4%. To ensure the calibrated sensor tips' reliability, we plan to limit their usage to a maximum of five days in this study. Considering the disposable nature of these sensor tips when applied in practical scenarios, we have determined that the fabricated sensor tips exhibit acceptable chemical stability in saliva for use in our smart mouthguard. In real‐time sensing, it is essential to evaluate the performance of continuous osmolar level tracking in response to concentration changes. The results of this continuous sensing and signal reproducibility are depicted in Figure 3C and the Figure S3A and B (Supporting Information). In gap electrode sensing, when analyzing transitions from high to low‐concentration samples, there is a potential for rebound signals from previously higher‐concentrated solutes. Nonetheless, Figure 3C indicates consistent reproducibility of admittance values in low osmolar samples, even after being immersed in higher osmolar solutions. The Figure S3A and B (Supporting Information) illustrate the changes in admittance due to continuous droplet insertion, causing incremental and decremental shifts in osmolar levels. The sensor tips were submerged in microtubes containing a specified osmolar level, and the continuous introduction of either 100 mOsm solution or deionized water elicited significant responsive changes in the sensor tip's admittance values. Additionally, the development process of smart mouthguards includes tests with osmometer calibration, artificial saliva, and real samples. Before advancing our sensor's continuous monitoring capability, a triplicated endpoint assay using the smart mouthguard's admittance measurement was performed on actual saliva samples. This study further validates the practical application and reliability of the sensor developed for the mouthguard. Saliva collection kits were utilized to gather saliva samples during 5 min of unstimulated secretion at various times: immediately upon waking, before a workout, and after 20 min of light exercise, such as jogging or walking. These collected samples were then centrifuged to separate residual substances, with the supernatant carefully extracted and applied to the sensing tips of the admittance analysis circuit on the mouthguard. As demonstrated in Figure S3C (Supporting Information), this process yielded stable and repeatable data, effectively illustrating the relationship between saliva osmolality changes before and after exercise. Overall, the linear calibration, chemical stability, and signal reproducibility of our fabricated sensor tips, along with the performance of the osmolality analysis circuit, have demonstrated promising proof‐of‐concept applicability for saliva analysis. However, to claim more accuracy, we conducted a comparison of our calibration study results with those obtained from a gold standard osmometer (using freezing depression) and commercial point‐of‐care (POC) testing devices (MX3) for saliva osmolality.^[^ ^47^ ^]^ The commercial POC device uses a disposable sensor, which is assumed to be an ion‐sensing electrode. Figure 3D shows three calibration curves from different devices, each proportional to the incremental level of calibration standards used (Table S1, Supporting Information). The smart mouthguard exhibited a high degree of linearity, with an R‐square of 0.997 and a calibration slope closer to the gold standard osmometer than that of the POC device, indicating superior accuracy compared to currently available commercial devices. Following the calibration study, saliva testing on human subjects was conducted. The saliva collection protocol, outlined in the kit, involved collecting samples before and after intermittent exercise. This process utilized a cotton swab placed in the mouth for 5 min, followed by centrifugation to extract cotton‐filtered saliva. These samples were then analyzed using a gold standard osmometer (Micro‐Osmette, Precision Systems Inc, Natick, MA), a commercial POC saliva test device (MX3), and a smart mouthguard. Additionally, body mass was tracked to correlate total water loss with changes in saliva osmolality as part of the endpoint assay. Pre‐ and post‐body mass measurement is a well‐known test for deducing body water content loss due to exertional activity. Athletes can assess pre‐ and post‐exercise body mass to accurately estimate sweat loss and evaluate hydration status. This practice facilitates the determination of fluid replacement requirements during and after exercise.^[^ ^48^, ^49^, ^50^ ^]^ Following this concept, we have set the training session for body mass loss tracking. The environmental condition was controlled in the room, and participants needed to follow strict rules for accurate test results. In detail, they asked not to consume food or water after 10 pm. Then, the pre‐ and post‐body mass measurement is accompanied by saliva osmolality tests. Table S2 (Supporting Information) presents the data on body mass loss and the increase in saliva osmolality from the samples collected during the exercise sessions. Each data point reflects the average measurements from the devices (with at least N = 3 for each). The participants' average age was 25.6 years, their average body mass was 85.3 kg, and their average resting heart rate was 82.3 bpm. For the treadmill sessions conducted in an environmental chamber, two separate running sessions were performed, and three saliva samples were collected from each. After the first and second sessions, body mass decreased by 1.5% and 2.8%, respectively. Another single cycling session resulted in a minimal 0.2% loss of body mass. A consistent incremental increase in osmolality was observed across all devices, including the gold‐standard osmometer, commercial device, and the smart mouthguard. In the tests on exercise‐involved human saliva samples, the smart mouthguard demonstrated over 91% accuracy compared to the gold standard osmometer. This finding is significant as it suggests that not only buffer and artificial saliva but also real samples from intense workout sessions can be effectively utilized for further evaluations. This result broadens the potential application and utility of the smart mouthguard in real‐world scenarios, particularly in monitoring hydration and health during physical activities. Furthermore, continuous monitoring of saliva osmolality can discern the case of drinking water, which results in the dilution of the substances in the mouth cavity for up to 15 min. This would be advantageous compared to the currently available POC devices, which result in only spot checks when the athlete is available for monitoring, typically during water breaks during practices or games. To further emphasize these advantages and enhance the device's reliability, we need to consider optimization involving collecting a larger number of real saliva samples in the future. These samples should be gathered under a variety of activities and across a broader range of dehydration conditions. The approaches mentioned will be designed to increase the applicability and accuracy of our device in real‐world scenarios.

### 2.4 Validation of the Bioelectric Signals on the Soft Chest Patch

**Figure 4:**
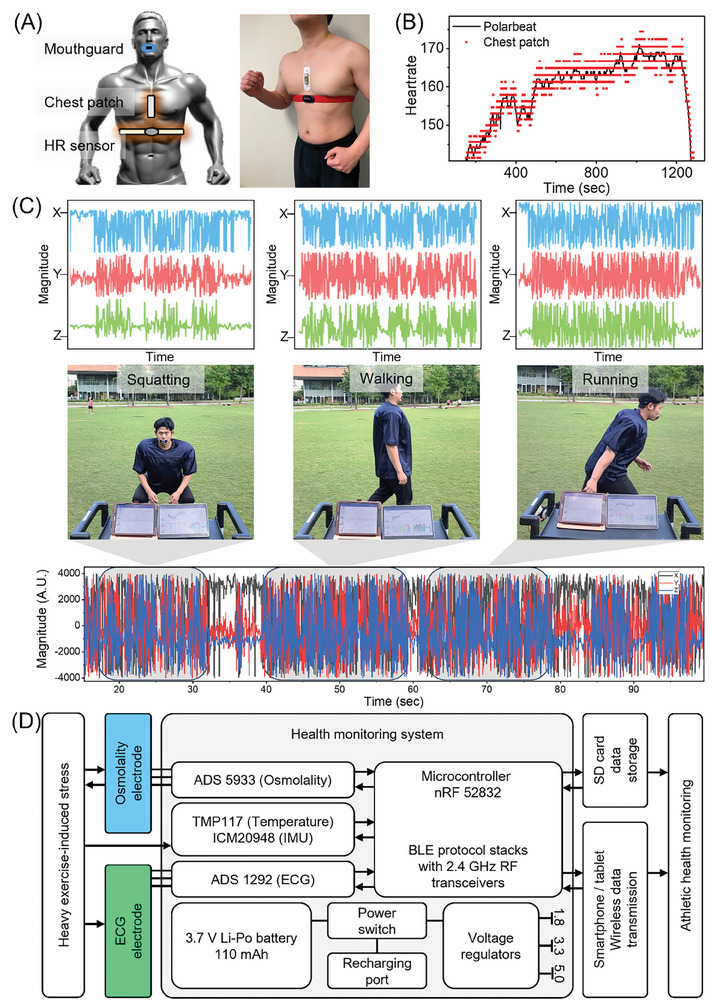
Validation of bioelectric signals from the soft chest patch. A) Schematic illustration showing device mounting locations (left) and a photo of a test subject wearing devices for an indoor treadmill test (right). B) Comparison data of heart rates between the soft chest patch and the commercial device (Polarbeat). C) Motion tracking data and specific movement charts; the top graphs show short‐term recorded accelerometer data for each activity, the middle photos show the test setup for continuous data monitoring using tablets, and the bottom graph displays a complete data set including all motions. D) Flowchart detailing data acquisition and processing using a mouthguard device and a chest patch.

The primary goal of the soft chest patch is to monitor ECG, skin temperature, and motion during exercise, facilitating real‐time data acquisition. Combining this patch with the smart mouthguard enhances the precision of detecting abnormal conditions during athletic activities, collectively offering a reliable assessment of an athlete's physiological health. For validation, the soft chest patch underwent testing during exercise. In‐lab testing involved evaluating target signal parameters in mild activity scenarios, including squatting, walking, and treadmill running. **Figure** **4**A shows the device configuration on a tester, incorporating the mouthguard, chest patch, and a commercial HR tracker to establish baseline applicability before extensive field testing. Previous studies have reported on the validations of chest patch devices; however, they did not include scenarios involving active motion.^[^ ^9^, ^44^ ^]^ Therefore, heat rate monitoring from a treadmill running test is conducted and its result is presented in Figure 4B. The study presents a chest patch used for physiological monitoring during a treadmill exercise, as shown in Figure S4A (Supporting Information). This patch is designed to measure HR, respiration rate, and temperature, with results from a 1600‐second test in Figure S4B (Supporting Information). The skin impedance from gold serpentine electrodes embedded in the medical tape is shown in Figure S4C (Supporting Information). While the skin impedance of the serpentine gold electrodes exhibited a higher range of impedance across the frequency spectrum than the Ag/AgCl gel electrode (Figure S4C, left, Supporting Information), their improved adhesive properties resulted in reduced skin impedance compared to previous studies. Specifically, at 100 Hz, an average impedance of 101.9 kΩ was measured in this study, whereas previous studies^[^ ^51^ ^]^ reported 153.6 kΩ (Figure S4C, right, Supporting Information). The ECG captured using this patch clearly delineates the P, QRS, and T waves, as well as the corresponding segments. The noise level between each segment was averaged, and maximum potential differences were used to calculate an average signal‐to‐noise ratio of 32.55 dB (Figure S4D, Supporting Information). The obtained signal‐to‐noise (SNR) value was recalculated following the equation for gold serpentine electrodes with a 0.5‐30 Hz bandpass filter, and below is utilized for SNR calculations (Equation 1).

(1) $$SNR\;\left(dB\right)=\;20\log\frac{RMS_{signal}}{RMS_{noise}}$$

where RMS_signal_ is the maximum root‐mean‐square value of the QRS complex and RMS_noise_ is the root‐mean‐square value of noise between the T‐ and P‐wave regions. All SNR values were collected over 2 min. We have added this information to the paragraph. However, we strongly agree with the reviewer's comment that SNR could be overestimated. We previously found that ECG readings can show high values due to existing sweat or remaining moisture.^[^ ^9^ ^]^ In some cases, participants had exceptionally high SNR values of 43.06 dB. To address this and ensure a reliable SNR ratio, we re‐evaluated the data with a longer monitoring time, increasing from 2 to 5 min, and increased the number of subjects from three (n = 3) to five (n = 5). The adjusted SNR ratio was calculated to be 32.55 dB. All data were collected during running at a speed of 10 km h^−1^. Despite collecting more samples, we still observed a high SNR ratio during exercise. We believe that the high SNR ratio during exercise is due to the sweat‐resistant strong adhesion and the strain‐separated backing layer. Figure S4E (Supporting Information) depicted that the calculated signal‐to‐noise ratio presents almost twice as high as previously reported ECG analysis in various positions using wearable devices. The patch's efficacy is partly due to the superior adhesive properties of the selected medical tapes and the encapsulation method. This combination ensures strong adhesion to the skin, allowing the adhesive substrate to maintain conformal contact with the electrodes, even during active movement of the torso. During this test, a commercial monitoring device from Polar (Polar, USA) recorded HR profiles and compared them with ECG data recorded and transmitted by the soft chest patches to a custom Android application. This necessitated the conversion of ECG data into HR readings. Figure 4B indicates a 90% match between the chest patch HR profile and that from the commercial tracker. Due to varying time frames in ECG analysis, moment‐to‐moment calculations of the chest patch showed broader ranges in short‐term periods. The chest patch's conformal contact and superior adhesive properties ensured undisturbed monitoring performance even at a running speed of 20 km h^−1^, unaffected by environmental factors like wind or impact from the ground. Subsequently, its motion‐tracking capabilities were evaluated outdoors. Considering the common use of mouthpieces with lip guards in American football, the tester wore the chest patch beneath football shoulder pads and shirts to assess motion tracking. Figure 4C displays three specific activities with corresponding postures and a photo of the tester and accelerometer graphs. The gyro sensor and accelerometer's 500‐second tracking record demonstrated continuous and sensitive tracking for each movement, with detailed activity profiles with the ECG and saliva osmolality tracking features captured in Video S1 (Supporting Information). Along with the three activities in the field, climbing up and down activities on the staircase was also demonstrated in the video. In conclusion, soft chest patches have proven effective for monitoring heart function, movement tracking, and skin temperature during exercise. Each device in this study harnesses wearable technology through designed circuitry comprising converters and various sensor components on the flexible substrate.

### 2.5 Integrated Smart Mouthguard and Soft Chest Patch System Overview

Figure 4D presents a detailed diagram illustrating the sensor data acquisition and processing flow for the smart mouthguards and the soft chest patches. The smart mouthguard features an osmolality sensing circuit utilizing an impedance converter (ADS 5933, Analog Devices). This component analyzes the admittance of saliva in contact with the micro‐gap electrode at a frequency of 100 Hz. The analyzed data is then transmitted to a multiprotocol system module (nRF 52 832, Nordic Semiconductor) for further processing. The soft chest patch uses gold serpentine electrodes to capture surface potential at 250 Hz for ECG monitoring. This data is fed into a front‐end analog‐to‐digital converter (ADC, ADS1292, Texas Instruments). Additionally, skin temperature and motion are tracked using digital temperature sensors (TMP117, Texas Instruments) and motion sensors (ICM‐20948, TDK Corporation), respectively. The collected data from all sensors is relayed to a multiprotocol system‐on‐chip module (nRF52832, Nordic Semiconductors). This module, equipped with an integrated microprocessor, not only receives sensor inputs but also oversees the functionality of the entire circuit. It processes the data and enables transmission to Bluetooth‐connected smartphones and tablets through a 2.4 GHz transceiver. A custom‐developed Android application installed on smartphones and tablets facilitates the continuous, real‐time display of all sensor data. Collectively, considering seamless data communication between the devices and the Android application, optimized sampling rate adjustment is required. Each sensor operates at a distinct sampling rate to facilitate the seamless transmission of comprehensive biosignal packages, including saliva osmolality, skin temperature, ECG, and its derivatives. The saliva osmolality measurement in the smart mouthguard, which shows minimal fluctuations on a minute scale, is sampled at 2 Hz. A higher sampling rate of 250 Hz is employed for ECG measurements to identify each cardiac peak accurately. HR calculations are derived from a 30‐second ECG data stack. This approach ensures efficient data communication without compromising signal integrity. The sampling rate for skin temperature monitoring is set at 3 Hz. Given the need to compute HR and temperature data for each sensor to derive the physiological strain value, the sampling rate for the finalized strain value is standardized at 2 Hz. This setup efficiently monitors an athlete's saliva osmolality, HR, temperature, and motion profiles. Additionally, the data is stored for subsequent analysis. In the event of Bluetooth connection failure, a backup storage system using an SD card is incorporated into each device, ensuring no data loss. For optimal performance and ease of use, all sensors and chip components are integrated into a flexible PCB, streamlining the operation of the devices in various athletic settings. **Table** **1** provides a comparative analysis of the proposed wearable system, focusing on its robust and multimodal sensing capabilities relative to prior studies. It also highlights the benefit of combining wearable systems positioned on different body parts, thereby broadening the ability to monitor specific aspects of human health, such as dehydration and cardiovascular strain. The table demonstrates a trend in health monitoring systems for exercise, showing a shift toward integrating general health metrics with targeted, multimodal sensing. This comparison with previously reported athletic devices, considering variables like specific target parameter monitoring and testing conditions, aids in evaluating the potential of our system for future applications in military/emergency personnel and athlete monitoring. Table 1 indicates that none of the existing devices simultaneously monitor continuous saliva and cardiovascular strain. Our devices, tested for functionality during active movements such as cycling, treadmill use, or field running, offer a feature not yet fully explored in previous models. The combination of saliva osmolality, ECG, temperature, and accelerometer readings in our devices allows for the potential of a detailed assessment of user status during intense exercise. Currently, wearable technologies mainly focus on multimodal sensing, primarily analyzing sweat‐based biomarkers or electrophysiological markers. Therefore, the integration of saliva osmolality monitoring with cardiovascular strain assessment in our devices could provide a unique, potentially more accurate validation method.

**Table 1:** Comparison of wearable devices’ performance in detecting activities of athletes.

Reference	Device type	Detection of continuous saliva osmolality	Types of measured cardiac signal	Types of activity during device testing
This work	Mouthguard and chest patch	Yes	ECG, HR, HRV	Running: 15–20 km h^−1^ Cycling: up to 40 km h^−1^
S. Imani et al. (2015)^[^ ^16^ ^]^	Chest patch	–	ECG, HR	Cycling
Camarillo et al. (2013)^[^ ^55^ ^]^	Mouthguard	–	–	Linear impactor: 7–28 km h^−1^
Kwon et al. (2020)^[^ ^25^ ^]^	Wrist band	–	HR	Running Punching
J. Park et al.(2017)^[^ ^56^ ^]^	Smart shirt	–	ECG, HR	–
Aguilar‐Torán et al. (2023)^[^ ^26^ ^]^	Chest band	–	HR	Cycling
Hedin et al. (2007)^[^ ^57^ ^]^	Mouthguard	–	–	Linear impactor: 10–40 km h^−1^
F. Sun et al. (2017)^[^ ^58^ ^]^	Smart shirt	–	ECG, HR	Running: 16 km h^−1^ Cycling
Faidah et al. (2020)^[^ ^47^ ^]^	Handheld type device	–	–	Static
M. Etemadi et al. (2017)^[^ ^59^ ^]^	Chest patch	–	ECG. SCG	Walking: 6.4 km h^−1^ Stair climbing: 5.5 km h^−1^
C. Shen et al. (2017)^[^ ^60^ ^]^	Smart shirt	–	ECG, RR	Running: 9 km h^−1^
T. Yamane et al. (2022)^[^ ^61^ ^]^	Smart Shirt	–	ECG	Running: 9 km h^−1^
T. Li et al. (2022)^[^ ^62^ ^]^	Chest patch	–	ECG	Cycling
Å. Ausland et al. (2022)^[^ ^29^ ^]^	Chest patch	–	ECG, HR	Running Poling Cycling
C. Qiu et al. (2022)^[^ ^63^ ^]^	Chest patch	–	RR	Running: 6 km h^−1^ Cycling: 20 km h^−1^
M. A. Zahed et al. (2023)^[^ ^64^ ^]^	Chest patch	–	ECG	Static
M. Sharifuzzaman et al. (2023)^[^ ^65^ ^]^	Chest patch	–	ECG	Cycling

### 2.6 Correlation of Saliva Osmolality and Cardiac Monitoring During Exercise

**Figure 5:**
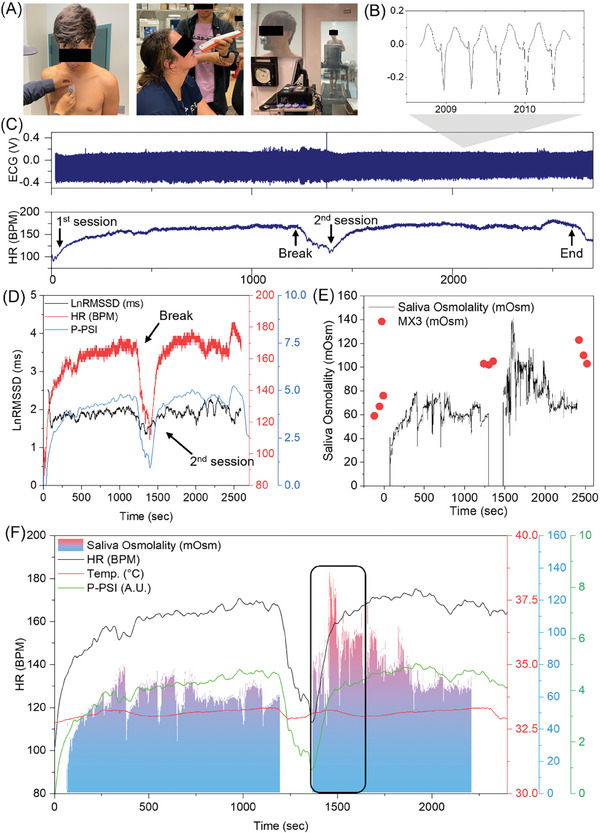
Experimental results of measured saliva osmolality and cardiovascular data from an hour‐long treadmill running test. A) Series of photos showing chest patch wear, saliva osmolality testing with a commercial device, and subject running on the treadmill. B) Magnified set of recorded ECG. C) Recorded ECG (top) and calculated HR from the ECG data (bottom). Each session timeline is depicted on the graph. D) Collective data of LnRMSSD HRV, HR, and P‐PSI. E) Comparison of measured saliva osmolality between a smart mouthguard showing continuous data measurement and a commercial device showing a discrete data detection (MX3). F) Collective set of continuously measured data including saliva osmolality, HR, temperature, and P‐PSI. The black box indicates the detected moment of the highest saliva osmolality peaks from the wearable devices.

While establishing the hypothesis of in‐depth dehydration assessment, we aimed to get an empirical data set of the direct and indirect biomarkers, saliva osmolality, and ECG derivate parameters from human subject testing protocol. However, the hypothesis regarding the specific dehydration‐associated event occurring point can only be assumed after the actual test. Thus, a long‐term (at least 40 min) data set of saliva osmolality, ECG, HR, HRV, and physiological strain parameters are prepared to check the correlated moment of dehydration or heat stress for the user. Physiological parameters can be affected by unintended external stimulations besides the exercise events, peripheral temperature, and humidity. Each parameter and derived calculations are carefully monitored to see the fluctuating tendencies. To see the correlated event, first tested on the controlled environmental chambers with specific activities confined to running and cycling. **Figure** **5**A shows photos of the preparation step for wearing a chest patch, commercial device testing for saliva osmolality, and testing sites in the environment‐controlled chamber. Video S2 (Supporting Information) captures the treadmill runner wearing a mouthguard and chest patch. Wireless data monitoring and recording were carried out using an Android tablet, with one of the test results illustrated in Figure 5B,C. The ECG feature (QRS) was identified from raw ECG data using a fast Fourier transform (Figure 5B). HR estimation, as shown in the lower part of Figure 5C, was derived from the processed ECG following a previously reported methodology.^[^ ^9^ ^]^ Heart rate is calculated by identifying the QRS complexes in the raw ECG data, typically through a process of thresholding to detect the R‐wave peaks, which correspond to individual heartbeats. Subsequently, HRV was determined using the natural logarithm of the root mean square of successive heartbeat differences (LnRMSSD). HRV, which reflects the variability between consecutive heartbeats, provides insights into human health conditions. In sports science, for instance, a gradual increase in average HRV in a resting state often correlates with enhanced aerobic performance, while a decrease may signal a heightened risk of injuries.^[^ ^52^ ^]^ Furthermore, another calculation for physiological strain index (PSI) from HR and temperature was conducted during exercise, indicating the heat strain on the body.^[^ ^53^ ^]^ PSI‐rated scales are 0 to 10 for heat‐related injury risk indication; generally utilized variables are rectal for core temperature and HR. In this study, we followed the PSI calculation from a prior study. However, due to the form factor of our device, we used non‐invasive skin temperature measurements as a proxy for core temperature instead of direct core temperature measurements. Therefore, the calculated index value would not perfectly match and exhibit the intended initial parameter. Nevertheless, we decided this value as an indirect identifying marker for heat stress during exercise.^[^ ^54^ ^]^ The adapted calculation consists of the following equation, namely pseudo‐PSI (P‐PSI), in this study (Equation 2).

(2) $$\begin{array}{ccc} \mathrm{pseudo}-\mathrm{PSI} & = & 5\left(T_{\mathrm{sk}}-T_{\mathrm{sk}0}\right)\cdot {\left(39.5--T_{\mathrm{sk}0}\right)}^{-1}\\ & & +5\left(\mathrm{HR}-HR_{0}\right)\cdot {\left(180-HR_{0}\right)}^{-1} \end{array}$$

where T_sk_ and HR are continuously monitored data of skin temperature and heart rate, and T_sk0_ and HR_0_ are the initial values before the exercise. From the calculation, 2600 sec of the monitored data of ECG and temperature was calculated and is depicted as HR, LnRMSSD HRV, and P‐PSI. In Figure 5D, the P‐PSI (blue line) showed fluctuations throughout the session, mirroring the HR trend (red line), especially noticeable during the break and at the onset of the second session, which induced an HR increase. However, LnRMSSD HRV (black line) demonstrated a less correlation with HR and P‐PSI trends before the break. From the 1400‐second in the second running session, the LnRMSSD curve exhibited an inversely proportional trend between 1600–2100 s and 2400–2550 s. Highlights that HRV, influenced by various human conditions, only occasionally correlates with other trends. Figure 5E outlines the limitations of the endpoint saliva osmolality assay. While continuous monitoring effectively captures dynamic fluctuations during exercise, the endpoint assay‐based POC device only reflects the overall trend. However, unlike in the static calibration study in Figure 3D, the current version of the saliva collection using the planar micro‐gap sensor structure still has limitations in reading sensitive changes of saliva osmolality within the oral cavity. As depicted in Figure 5E’s continuous data, the dataset fluctuates multiple times within a few seconds. This fluctuation is due to the random contact events between the tongue and the sensing surface. The complex surface of the tongue, with its papillae and sublingual skin, easily affects the admittance fluctuation when contact occurs. Nevertheless, fresh saliva delivery to the sensor tip necessarily requires the natural random movement of the tongue in the oral cavity. Therefore, modifications to the sensing tip, such as a micro‐structured porous lid to selectively intake fluid and prevent contact events, will be designed to avoid fluctuating data during analysis in the next works. Figure 5F represents the whole running session monitoring data of saliva osmolality, HR, skin temperature, and P‐PSI data. Between 1480 and 1770 s, saliva osmolality increased from 60 to 120 mOsm, suggesting mild dehydration after ≈300 s. Concurrently, HR and P‐PSI also rose from 113 to 168 BPM (148% increase) and 0.98 to 4.38 (440% increase), respectively. Skin temperature marginally increased from 32.9 to 33.2 °C but decreased again within 120 s. These variations indicate that acute mild dehydration may lead to exercise‐induced physiological strain. The correlation between these metrics suggests that specific time windows showing acute changes could be crucial for assessing dehydration.

### 2.7 Field Test for Dehydration and Heat Strain Monitoring

**Figure 6:**
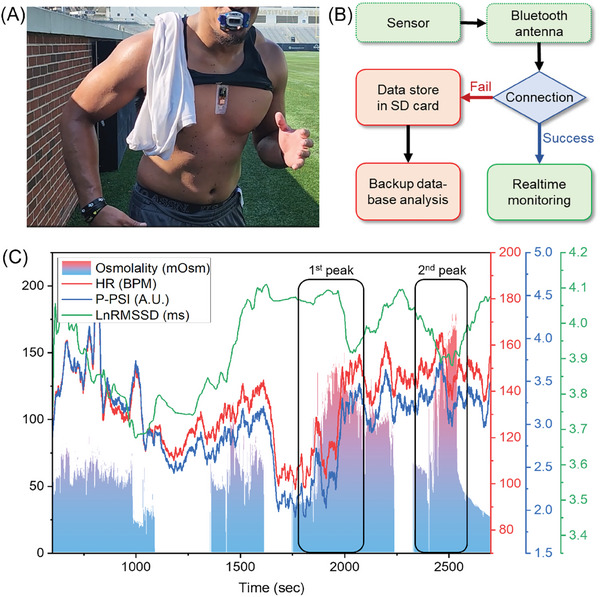
Field test during a football players' training session. A) Photo of a football player wearing a smart mouthguard and a soft chest patch. B) Flow chart showing backup storage implementation in the system. The measured data can be automatically recorded to the microSD when Bluetooth connection between devices occurs. C) Collective graph displaying recorded saliva osmolality, HR, P‐PSI, and LnRMSSD HRV.

To evaluate the practical application of the smart mouthguard and soft chest patch, football players from Georgia Tech use these devices during a training session at the stadium. **Figure** **6**A shows the device‐wearing setting for the test. Soft chest patches were distributed before training, and smart mouthguards were provided after an hour‐long session. A particular test for the smart mouthguard involved subjects who did not consume water during the session. In field training scenarios where players are likely to move beyond the range of Bluetooth communication (up to 10 meters), we activated the SD card backup protocol to the devices to ensure continuous data collection. A protocol in the firmware initiates recording to the SD card if the Bluetooth connection to the Android device is lost, as illustrated in Figure 6B. The training session consisted of repetitive Burpee tests, sprinting, and frequent recovery breaks, and our devices recorded and monitored the target parameters. Figure 6C presents a matched data set ranging from 600 to 2700 s of saliva osmolality, HR, P‐PSI, and HRV. Among the data, the saliva osmolality readings intermittently showed blank due to the momentary taking off the device during the short break. On the other hand, the chest patches continuously recorded ECG and temperature data throughout the session. Prior to the sign of exercise‐elicited dehydration via saliva osmolality, HR and P‐PSI exhibited gradual fluctuations in response to exercise and short break periods, as illustrated in Figure 6C. Specifically, before the 1100‐second mark, given that the session involving the smart mouthguard had been active after an hour of warming up, HR and P‐PSI began with elevated levels, then declined until the short breaks. Concurrently, saliva osmolality remained below the fully hydrated threshold of 75 mOsm. However, following the second break at 1750 s, identified as the first peak event, there was a notable escalation in HR (from 103 to 154 BPM, 149% increase) and P‐PSI (from 2% to 3.5%, 175% increase), reaching a maximum at 2060 s. This increase coincided with a spike in saliva osmolality from 37 to 140 mOsm, reflecting a correlated trend with HR and P‐PSI. Similarly, during the second peak event, ≈2500 s, HR and P‐PSI continued their upward trend, paralleling the increases in saliva osmolality. The combined use of the smart mouthguard and soft chest patches has proven successful in collecting data, capturing both the moderately dehydrated state of the user and changes in the correlated physiological stress index. However, further research is needed to assess the impact of immediate water intake during training and refine the calculation of the physiological strain index based on skin temperature. This requires additional data for a more accurate assessment of strain levels. Further, a countermeasure protocol is necessary for water or sports drink intake while testing is ongoing. For now, we will have set the rejoining protocols, including washing the oral cavity with distilled water, resting for 15 min, and then getting back to monitoring. However, instant recalibration of saliva osmolality can be imagined for future applications. Additionally, while saliva osmolality is a helpful indicator of hydration levels, it may lack the sensitivity to detect severe dehydration. Despite this, the use of these wearable devices in tandem shows promise for issuing alerts about acute to moderate dehydration. Continued research in this area is essential to understand better the complexities and dynamic nature of real‐time physiological fluid analysis.

## 3 Conclusion

This paper introduces an all‐in‐one wearable system capable of monitoring athlete health during training sessions. This wearable sensor package integrates ultrathin flexible sensors and electronics with an athlete's mouthguard and chest patch. The primary function of a sensor‐integrated mouthguard is to monitor saliva osmolality for continuous dehydration detection. A soft chest patch monitors heart functions, skin temperature, and activity levels during exercise, enabling real‐time data collection. Simultaneously, it facilitates the estimation of HR and physiological strain from ECG and temperature sensors, which plays a role in identifying exercise‐induced heat strain. The integrated wearable system has successfully recorded field test data, revealing moments when HR, physiological strain, and saliva osmolality are correlated. This combination delivers critical data on heat stress and dehydration detection, complemented by saliva osmolality measurements, thus offering an early warning system for physiological strain linked to acute dehydration events. Collectively, the wearable sensor package presented in this work shows a promise for advancing the healthcare of athletes and military/emergency personnel operating in extreme environments.

## 4 Experimental Section

### Materials and Apparatus

The osmolality sensor calibration utilized standard osmotic solutions from Precision Systems Inc., USA. For validation, a gold standard osmometer (Micro‐Osmette, Precision Systems Inc., Natick MA) was used for freezing‐point depression measures of solutions. Artificial saliva (product number 1700‐0316) was sourced from Pickering Scientific, USA. The point‐of‐care saliva test device was acquired from MX3 hydration testing system‐Pro version (MX3 Diagnostics, Melbourne, Australia). An HR strap tracking device was procured from Polar, USA. EcoFlex 00–30 for device encapsulation was obtained from Smooth‐On, USA. The heat‐responsive polyimide film, Pyralux, was purchased from DuPont, USA. Various medical tapes for testing purposes were supplied by 3 M, USA. Football shoulder pads were provided by Georgia Tech Athletic Association collegiate team. The impedance converter ADS 5933 was purchased from Analog Devices. The multiprotocol system module nRF 52 832 was sourced from Nordic Semiconductor. The front‐end analog‐to‐digital converter ADS1292 and digital temperature sensors TMP117 were manufactured by Texas Instruments. The motion sensor ICM‐20948 was obtained from TDK Corporation.

### Preparation of Osmolality Sensor

A clean aluminum substrate, measuring 3×5 inches, was spin‐coated with polydimethylsiloxane (PDMS, Sylgard 184, Dow) using a ratio of 1:5 for the curing agent to base mixture. Then, copper foil was laminated onto the PDMS‐coated aluminum substrate and cleaned with isopropanol. The copper‐laminated substrate and a heat‐responsive polyimide sheet were both patterned with 100‐micrometer gap electrodes and their corresponding laminating covers using femtosecond laser micromachining (Optec laser systems, Belgium). To complete the encapsulation process while leaving the copper micro‐gap pattern exposed, a heat press was applied at 350 °C for 20 min. Subsequently, a commercial gold electroplating solution was employed at 3 V and 0.1 mA for 30 s to deposit a gold layer over the exposed copper areas. Finally, a laminated copper wire with a diameter of 0.3 mm was connected to both the impedance converter‐equipped circuit and the sensing tips, finalizing the assembly.

### Integration of Smart Mouthguard

A commercial mouthguard (Shock Doctor, USA) featuring an interchangeable mouthpiece and lip guard was modified to incorporate the admittance sensing circuit and a Li‐Po battery. The lip guard surface was cleaned using acetone and isopropanol to remove any existing coating layers. Then, epoxy (Loctite) was applied to the treated surface to secure the circuitry and battery. An elastomer mix was prepared to protect and encapsulate the entire exposed area of the circuitry, including the lip guard surface. This mix used Ecoflex GEL 00–30 (Smooth‐On), blended in a 1:1 ratio of curing agent to base. This encapsulation ensures the durability and functionality of the integrated components within the mouthguard.

### Preparation of Chest Patch Electrodes

A clean glass slide was prepared by spin‐coating it with PDMS (Sylgard 184, Dow), using a 1:10 ratio of curing agent to base. Subsequently, a 12.7 µm thick polyimide sheet (50HN Kapton Film, DuPont) was laminated onto the PDMS layer. Cr (Chromium) and Au (Gold) layers were then deposited onto this laminate, with thicknesses of 5 and 200 nm, respectively, utilizing an electron beam deposition tool (Denton Explorer). For patterning the metal‐Kapton laminate, a femtosecond IR laser micromachining tool (Optec) was employed, set at a laser power of 50 W and with two cycles of pattern repetition. After removing the nonfunctional portions, water‐soluble tape (AQUASOL) was used to transfer the functional part from the PDMS/glass substrate onto the adhesive side of a 4076 patch. Following the removal of the water‐soluble tape and drying the sample, a low‐modulus elastomer (Ecoflex GEL, Smooth‐On) was applied along the interconnect lines. This was then left to cure at room temperature for 1 h, providing insulation while maintaining the overall adhesiveness of the assembly. This method ensures that the functional integrity of the component was preserved while also providing the necessary insulation and adhesive properties.

### Human Subject Testing in Environmental Chamber

The human pilot study was conducted with the participation of several healthy volunteers, adhering to the IRB‐approved protocol (#H23170) from the Georgia Institute of Technology. All participants provided their informed consent by signing the requisite forms, thereby authorizing the experimental procedures. Before participating in the test, subjects were required to abstain from eating and drinking (including water) after their evening meal. Upon arrival in the morning at the test location, nude body mass was initially recorded. Saliva collection was conducted using a cotton swab kit for a duration of 5 min. Concurrently, a soft chest patch was attached to the subject's upper sternum. Once the Bluetooth connection was verified as active, subjects entered a hot environmental chamber (30 °C, 50% relative humidity). The treadmill and cycling consisted of two sessions and a 5 min break after 20 min of the first session at a self‐selected pace. During the session, the speed range of the treadmill and cycling was 15 to 20 and 40 km h^−1^. Immediately after the session concluded, any sweat on the skin was wiped off with a towel, and the subjects' nude body mass measured again. This was followed by another round of cotton swab‐based saliva collection. The collected saliva samples were centrifuged for 10 min and supernatant aliquoted for osmolality assessment using a gold standard osmometer, commercial POC saliva test device, and smart mouthguard.

## Conflict of Interest

The authors declare no conflict of interest.

## Author Contributions

K.R.K. and T.W.K. contributed equally to this work. K.R.K, T.W.K., S.H.L., H.S.K., H.K., M.M.‐S., and W.‐H.Y. conceptualized the work. K.R.K, T.W.K., H.K., J.M., H.K., and M.M.‐S performed the experiments. K.R.K., T.W.K., H.K., Y.J.L., and H.K. analyzed the data. K.R.K., T.W.K., M.M.‐S, and W.‐H.Y. wrote the paper.

## Supporting information

Supporting Information

Supplemental Video 1

Supplemental Video 2

## Data Availability

The data that support the findings of this study are available from the corresponding author upon reasonable request.

## References

[1] H. C. Ates , P. Q. Nguyen , L. Gonzalez‐Macia , E. Morales‐Narváez , F. Güder , J. J. Collins , C. Dincer , Nat. Rev. Mater. 2022, 7, 887. 35910814 10.1038/s41578-022-00460-x PMC9306444

[2] H. Kim , Y. J. Lee , G. Byun , C. Choi , W.‐H. Yeo , Adv. Electron. Mater. 2023, 9, 2201294.

[3] H.‐R. Lim , H. S. Kim , R. Qazi , Y.‐T. Kwon , J.‐W. Jeong , W.‐H. Yeo , Adv. Mater. 2020, 32, 1901924. 10.1002/adma.201901924 31282063

[4] T. Strain , K. Wijndaele , P. C. Dempsey , S. J. Sharp , M. Pearce , J. Jeon , T. Lindsay , N. Wareham , S. Brage , Nat. Med. 2020, 26, 1385. 32807930 10.1038/s41591-020-1012-3 PMC7116559

[5] S. Pham , D. Yeap , G. Escalera , R. Basu , X. Wu , N. J. Kenyon , I. Hertz‐Picciotto , M. J. Ko , C. E. Davis , Sensors 2020, 20, 855. 32041097 10.3390/s20030855 PMC7039288

[6] A. Kristoffersson , M. Lindén , Sensors 2022, 22, 573. 35062531 10.3390/s22020573 PMC8778538

[7] M. L. Hoang , M. Carratù , V. Paciello , A. Pietrosanto , Sensors 2021, 21, 2313. 33810301 10.3390/s21072313 PMC8036345

[8] H. Kim , Y. J. Yoo , J. H. Yun , S.‐Y. Heo , Y. M. Song , W.‐H. Yeo , Adv. Healthcare Mater. 2023, 12, 2301104. 10.1002/adhm.202301104 37548604

[9] Y. S. Kim , J. Kim , R. Chicas , N. Xiuhtecutli , J. Matthews , N. Zavanelli , S. Kwon , S. H. Lee , V. S. Hertzberg , W. H. Yeo , Adv. Healthcare Mater. 2022, 11, e2200170. 10.1002/adhm.202200170 PMC9262812 35306761

[10] Y. Liu , A. F. McGuire , H. Y. Lou , T. L. Li , J. B. Tok , B. Cui , Z. Bao , Proc. Natl. Acad. Sci. U S A 2018, 115, 11718. 30377271 10.1073/pnas.1810827115 PMC6243254

[11] C. Xu , Y. Song , J. R. Sempionatto , S. A. Solomon , Y. Yu , H. Y. Y. Nyein , R. Y. Tay , J. Li , W. Heng , J. Min , A. Lao , T. K. Hsiai , J. A. Sumner , W. Gao , Nat. Electron. 2024. 10.1038/s41928-023-01116-6 PMC10906959 38433871

[12] H. R. Lim , S. M. Lee , M. Mahmood , S. Kwon , Y. S. Kim , Y. Lee , W. H. Yeo , Sensors (Basel) 2021, 21. 33652955 10.3390/s21051642 PMC7956447

[13] L. Garcia‐Carmona , A. Martin , J. R. Sempionatto , J. R. Moreto , M. C. Gonzalez , J. Wang , A. Escarpa , Anal. Chem. 2019, 91, 13883. 31573188 10.1021/acs.analchem.9b03379

[14] M. Wang , Y. Yang , J. Min , Y. Song , J. Tu , D. Mukasa , C. Ye , C. Xu , N. Heflin , J. S. McCune , T. K. Hsiai , Z. Li , W. Gao , Nat. Biomed. Eng. 2022, 6, 1225. 35970928 10.1038/s41551-022-00916-z PMC10432133

[15] A. Nguyen , R. Alqurashi , Z. Raghebi , F. Banaei‐kashani , A. C. Halbower , T. Vu , in Proceedings of the 14th ACM Conference on Embedded Network Sensor Systems CD‐ROM , 2016.

[16] S. Imani , A. J. Bandodkar , A. M. V. Mohan , R. Kumar , S. Yu , J. Wang , P. P. Mercier , Nat. Commun. 2016, 7, 11650. 27212140 10.1038/ncomms11650 PMC4879240

[17] P. Dongiovanni , M. Meroni , S. Casati , R. Goldoni , D. V. Thomaz , N. S. Kehr , D. Galimberti , M. Del Fabbro , G. M. Tartaglia , Int. J. Oral Sci. 2023, 15, 27. 37386003 10.1038/s41368-023-00231-6 PMC10310701

[18] J. Hua , L. Tian , Stat. Methods Med. Res. 2021, 30, 1101. 33522437 10.1177/0962280220987587

[19] M. R. Mamtani , T. P. Thakre , M. Y. Kalkonde , M. A. Amin , Y. V. Kalkonde , A. P. Amin , H. Kulkarni , BMC Bioinformatics 2006, 7, 442. 17032455 10.1186/1471-2105-7-442 PMC1618410

[20] T. Xu , Y. Fang , A. Rong , J. Wang , BMC Med. Res. Methodol. 2015, 15, 94. 26521228 10.1186/s12874-015-0085-z PMC4628350

[21] T. Arakawa , K. Tomoto , H. Nitta , K. Toma , S. Takeuchi , T. Sekita , S. Minakuchi , K. Mitsubayashi , Anal. Chem. 2020, 92, 12201. 32927955 10.1021/acs.analchem.0c01201

[22] W. Hong , W. G. Lee , Analyst 2020, 145, 7796. 10.1039/d0an01484b 33107873

[23] H. Mirzajani , F. Mirlou , E. Istif , R. Singh , L. Beker , Biosens. Bioelectron. 2022, 197, 113761. 34800926 10.1016/j.bios.2021.113761

[24] H. Seo , W. G. Chung , Y. W. Kwon , S. Kim , Y.‐M. Hong , W. Park , E. Kim , J. Lee , S. Lee , M. Kim , K. Lim , I. Jeong , H. Song , J.‐U. Park , Chem. Rev. 2023, 123, 11488. 37748126 10.1021/acs.chemrev.3c00290 PMC10571045

[25] S. Kwon , Y. T. Kwon , Y. S. Kim , H. R. Lim , M. Mahmood , W. H. Yeo , Biosens. Bioelectron. 2020, 151, 111981. 31999588 10.1016/j.bios.2019.111981

[26] J. Aguilar‐Toran , G. Rabost‐Garcia , S. Toinga‐Villafuerte , A. Alvarez‐Carulla , V. Colmena‐Rubil , A. Fajardo‐Garcia , A. Cardona‐Bonet , J. Casals‐Terre , X. Munoz‐Pascual , P. Miribel‐Catala , J. Punter‐Villagrasa , Sensors (Basel) 2023, 23. 38067846 10.3390/s23239473 PMC10708619

[27] W.‐H. Yeo , Y.‐S. Kim , J. Lee , A. Ameen , L. Shi , M. Li , S. Wang , R. Ma , S. H. Jin , Z. Kang , Y. Huang , J. A. Rogers , Adv. Mater. 2013, 25, 2773. 23440975 10.1002/adma.201204426

[28] Y.‐T. Kwon , Y. Lee , G. K. Berkmen , H.‐R. Lim , L. Scorr , H. A. Jinnah , W.‐H. Yeo , Adv. Mater. Technol. 2019, 4, 1900458. 33043125 10.1002/admt.201900458 PMC7546326

[29] Å. Ausland , E. L. Sandberg , J. Jortveit , S. Seiler , Front. Sports and Active Living 2022, 4. 10.3389/fspor.2022.937525 PMC9357913 35958669

[30] B. P. McDermott , S. A. Anderson , L. E. Armstrong , D. J. Casa , S. N. Cheuvront , L. Cooper , W. L. Kenney , F. G. O'Connor , W. O. Roberts , J. Athletic Training 2017, 52, 877. 10.4085/1062-6050-52.9.02 PMC5634236 28985128

[31] M. T. Wittbrodt , M. Millard‐Stafford , Med. Sci. Sports Exerc 2018, 50, 2360. 29933347 10.1249/MSS.0000000000001682

[32] J. A. Owen , M. B. Fortes , S. Ur Rahman , M. Jibani , N. P. Walsh , S. J. Oliver , Int. J. Sport Nutr. Exercise Metab. 2019, 29, 604. 10.1123/ijsnem.2019-0022 31141419

[33] S. N. Cheuvront , R. W. Kenefick , E. J. Zambraski , Int. J. Sport Nutr. Exercise Metab. 2015, 25, 293. 10.1123/ijsnem.2014-0138 25386829

[34] S. N. Cheuvront , C. X. Muñoz , R. W. Kenefick , Am. J. Clin. Nutr. 2016, 104, 553. 27465376 10.3945/ajcn.115.129858

[35] T. Hew‐Butler , C. Eskin , J. Bickham , M. Rusnak , M. VanderMeulen , Medicine & Science in Sports & Exercise 2018, 50, 341. 10.1136/bmjsem-2017-000297 PMC5812394 29464103

[36] D. L. Smith , I. Shalmiyeva , J. DeBlois , M. Winke , Prehospital Emergency Care 2012, 16, 128. 21950414 10.3109/10903127.2011.614044

[37] C. X. Muñoz , E. C. Johnson , J. K. DeMartini , R. A. Huggins , A. L. McKenzie , D. J. Casa , C. M. Maresh , L. E. Armstrong , Eur. J. Clin. Nutri. 2013, 67, 1257. 10.1038/ejcn.2013.195 24129362

[38] B. Sperlich , K. Aminian , P. Düking , H.‐C. Holmberg , Front. Physio. 2020, 10. 10.3389/fphys.2019.01520 PMC6960165 31969826

[39] C.‐H. Chen , Y.‐P. Lu , A.‐T. Lee , C.‐W. Tung , Y.‐H. Tsai , H.‐P. Tsay , C.‐T. Lin , J.‐T. Yang , J. Personal. Med. 2021, 11, 577.

[40] L. E. Armstrong , J. Am. College of Nutri. 2007, 26, 575S. 10.1080/07315724.2007.10719661 17921468

[41] A. R. Hand , in Dental Science for the Medical Professional: An Evidence‐Based Approach, (Eds: C. E. Niekrash , E. M. Ferneini , M. T. Goupil ), Springer International Publishing, Cham 2023.

[42] N. A. S. Taylor , A. M. J. van den Heuvel , P. Kerry , S. McGhee , G. E. Peoples , M. A. Brown , M. J. Patterson , Eur. J. Appl. Physiol. 2012, 112, 3227. 22230919 10.1007/s00421-011-2299-z

[43] S. Kwon , H. S. Kim , K. Kwon , H. Kim , Y. S. Kim , S. H. Lee , Y.‐T. Kwon , J.‐W. Jeong , L. M. Trotti , A. Duarte , W.‐H. Yeo , Sci. Adv. 2023, 9, eadg9671. 37224243 10.1126/sciadv.adg9671 PMC10208583

[44] J. Kim , P. Kantharaju , H. Yi , M. Jacobson , H. Jeong , H. Kim , J. Lee , J. Matthews , N. Zavanelli , H. Kim , H. Jeong , M. Kim , W.‐H. Yeo , npj Flexible Electron. 2023, 7, 3.

[45] N. Rodeheaver , H. Kim , R. Herbert , H. Seo , W.‐H. Yeo , ACS Appl. Electro. Mater. 2022, 4, 503.

[46] S. N. Cheuvront , R. W. Kenefick , K. R. Heavens , M. G. Spitz , J. Clin. Lab. Anal. 2014, 28, 368. 24648281 10.1002/jcla.21695 PMC6807512

[47] N. Faidah , G. V. Soraya , M. Erlichster , R. Natzir , G. Chana , E. Skafidas , M. Hardjo , I. J. Ganda , B. Bahar , Journal of Paediatrics and Child Health 2021, 57, 813. 33373495 10.1111/jpc.15325

[48] R. J. Maughan , S. M. Shirreffs , Scandinavian J. Medic. Sci. Sports 2010, 20, 40. 10.1111/j.1600-0838.2010.01207.x 21029189

[49] G. Harvey , R. Meir , L. Brooks , K. Holloway , J. Medic. Sci. Sports 2008, 11, 600. 10.1016/j.jsams.2007.05.012 17888734

[50] L. B. Baker , J. A. Lang , W. L. Kenney , Eur. J. Appl. Physiol. 2009, 105, 959. 19156437 10.1007/s00421-009-0982-0

[51] H. Kim , Y.‐S. Kim , M. Mahmood , S. Kwon , N. Zavanelli , H. S. Kim , Y. S. Rim , F. Epps , W.‐H. Yeo , Adv. Sci. 2020, 7, 2000810. 10.1002/advs.202000810 PMC7404159 32775164

[52] A. E. Aubert , B. Seps , F. Beckers , Sports Medicine 2003, 33, 889. 12974657 10.2165/00007256-200333120-00003

[53] D. S. Moran , A. Shitzer , K. B. Pandolf , Am. J. Physiol.: Regul., Integr. Comp. Physiol. 1998, 275, R129. 10.1152/ajpregu.1998.275.1.R129 9688970

[54] J. S. Cuddy , M. Buller , W. S. Hailes , B. C. Ruby , Military Medicine 2013, 178, e841. 23820362 10.7205/MILMED-D-12-00524

[55] D. B. Camarillo , P. B. Shull , J. Mattson , R. Shultz , D. Garza , Ann. Biomed. Eng. 2013, 41, 1939. 23604848 10.1007/s10439-013-0801-y PMC3954756

[56] J.‐A. Park , H.‐J. Han , J.‐C. Heo , J.‐H. Lee , Computer Assisted Surgery 2017, 22, 176. 29037055 10.1080/24699322.2017.1389396

[57] D. S. Hedin , P. L. Gibson , A. J. Bartsch , S. Samorezov , presented at 2016 38th Annual International Conference of the IEEE Engineering in Medicine and Biology Society (EMBC) , 16–20 Aug. 2016, 2016. 10.1109/EMBC.2016.7591119 28268724

[58] F. Sun , C. Yi , W. Li , Y. Li , Computers in Industry 2017, 1.

[59] M. Etemadi , O. T. Inan , J. Appl. Physiol. 2018, 124, 452. 28798198 10.1152/japplphysiol.00298.2017 PMC5867366

[60] C.‐L. Shen , T.‐H. Huang , P.‐C. Hsu , Y.‐C. Ko , F.‐L. Chen , W.‐C. Wang , T. Kao , C.‐T. Chan , J. Med. Bio. Engineer. 2017, 37, 826. 10.1007/s40846-017-0247-z PMC6132375 30220900

[61] T. Yamane , K. Hirano , K. Hirai , D. Ousaka , N. Sakano , M. Morita , S. Oozawa , S. Kasahara , Adv. Biomed. Eng. 2022, 11, 151.

[62] T. Li , B. Liang , Z. Ye , L. Zhang , S. Xu , T. Tu , Y. Zhang , Y. Cai , B. Zhang , L. Fang , X. Mao , S. Zhang , G. Wu , Q. Yang , C. Zhou , X. Cai , X. Ye , Biosens. Bioelectron. 2022, 198, 113855. 34871834 10.1016/j.bios.2021.113855

[63] C. Qiu , F. Wu , W. Han , M. R. Yuce , IEEE Trans. Biomed. Eng. 2022, 69, 2970. 35275808 10.1109/TBME.2022.3158544

[64] M. A. Zahed , D. K. Kim , S. H. Jeong , M. Selim Reza , M. Sharifuzzaman , G. B. Pradhan , H. Song , M. Asaduzzaman , J. Y. Park , ACS Sens. 2023, 8, 2960. 37498214 10.1021/acssensors.3c00148

[65] M. Sharifuzzaman , M. A. Zahed , M. S. Reza , M. Asaduzzaman , S. Jeong , H. Song , D. K. Kim , S. Zhang , J. Y. Park , Adv. Funct. Mater. 2023, 33, 2208894.

