# Supplementary material for: All‐in‐One, Wireless, Multi‐Sensor Integrated Athlete Health Monitor for Real‐Time Continuous Detection of Dehydration and Physiological Stress
Source: Adv Sci (Weinh). 2024 Jul 1;11(33):2403238. doi: 10.1002/advs.202403238 (PMC11434103; doi:10.1002/advs.202403238)
Supplement: Supplementary file 1 — Supporting Information [file ADVS-11-2403238-s001.pdf]

ADVANCED SCIENCE
Open Access

Supporting Information

for *Adv. Sci.*, DOI 10.1002/advs.202403238

# All-in-One, Wireless, Multi-Sensor Integrated Athlete Health Monitor for Real-Time Continuous Detection of Dehydration and Physiological Stress

*Ka Ram Kim, Tae Woog Kang, Hodam Kim, Yoon Jae Lee, Sung Hoon Lee, Hoon Yi, Hyeon Seok Kim, Hojoong Kim, Jihee Min, Jud Ready, Melinda Millard-Stafford and Woon-Hong Yeo**

**Supporting Information**

**All-in-One, Wireless, Multi-Sensor Integrated Athlete Health Monitor for Real-Time Continuous Detection of Dehydration and Physiological Stress**

*Ka Ram Kim, Tae Woog Kang, Hodam Kim, Yoon Jae Lee, Sung Hoon Lee, Hoon Yi, Hyeon Seok Kim, Hojoong Kim, Jihee Min, Jud Ready, Melinda Millard-Stafford, and Woon-Hong Yeo**

Dr. K. R. Kim, Dr. T. W. Kang, Dr. H. Kim, Dr. H. Yi, Dr. H. S. Kim, Dr. H. Kim, and Prof. W. -H. Yeo
George W. Woodruff School of Mechanical Engineering, College of Engineering, Georgia Institute of Technology, Atlanta, GA 30332, USA
Dr. K. R. Kim, Dr. T. W. Kang, Dr. H. Kim, Y. J. Lee, Dr. S. H. Lee, Dr. H. Yi, Dr. H. S. Kim, Dr. H. Kim, J. Min, and Prof. W. -H. Yeo
Center for Wearable Intelligent Systems and Healthcare, Institute for Matter and Systems, Georgia Institute of Technology, Atlanta, GA 30332, USA
Y. J. Lee and Dr. S. H. Lee
School of Electrical and Computer Engineering, Georgia Institute of Technology, Atlanta, Georgia 30332, USA
J. Min
Department of Biology, College of Arts and Sciences, Emory University, Atlanta, Georgia 30322, USA
Dr. Jud Ready
Electro-Optical Systems Laboratory, Georgia Tech Research Institute, Atlanta, Georgia 30332, USA
Dr. M. Millard-Stafford
School of Biological Sciences, Georgia Institute of Technology, Atlanta, Georgia 30332, USA
Dr. W. -H. Yeo
Wallace H. Coulter Department of Biomedical Engineering, Georgia Tech and Emory University School of Medicine, Atlanta, Georgia 30332, USA
Dr. W. -H. Yeo
Parker H. Petit Institute for Bioengineering and Biosciences, Institute for Robotics and Intelligent Machines, Georgia Institute of Technology, Atlanta, Georgia 30332, USA
*E-mail: whyeo@gatech.edu (W. -H. Yeo)

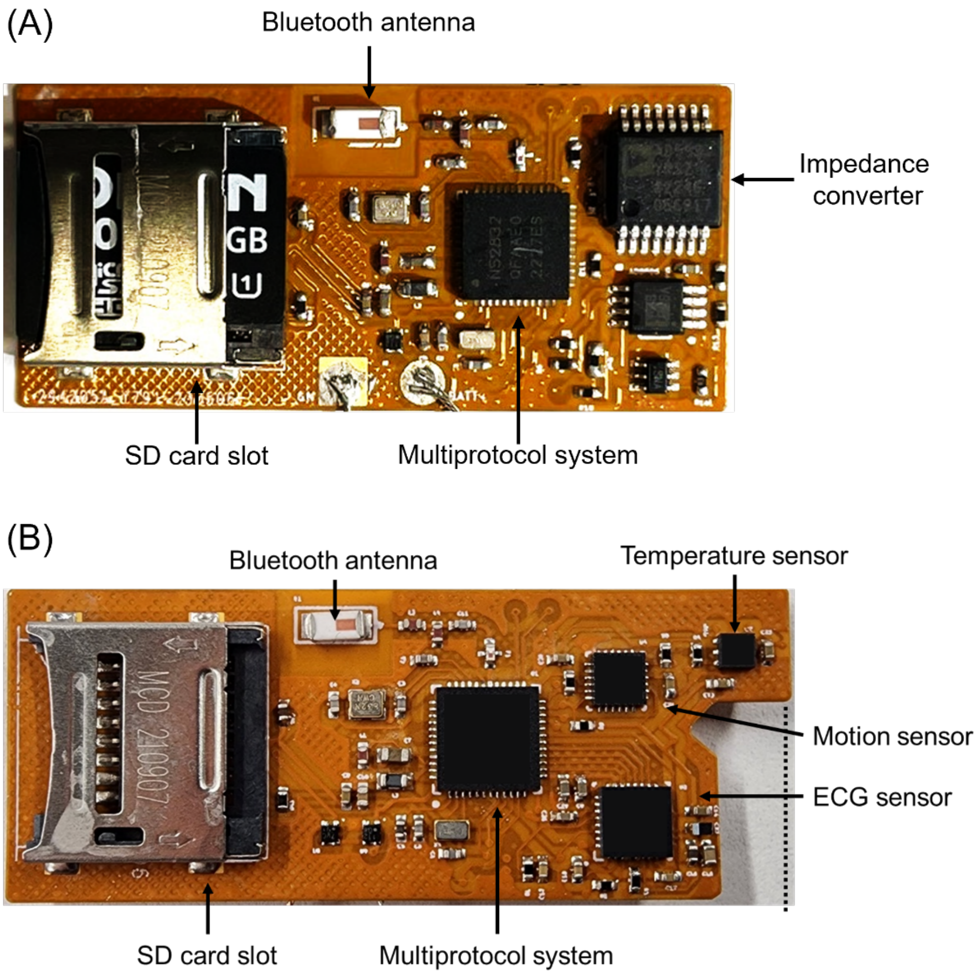


**Figure S1. Circuitry design of the developed wearable devices.** (A) Smart mouthguard. (B) Soft chest patch.

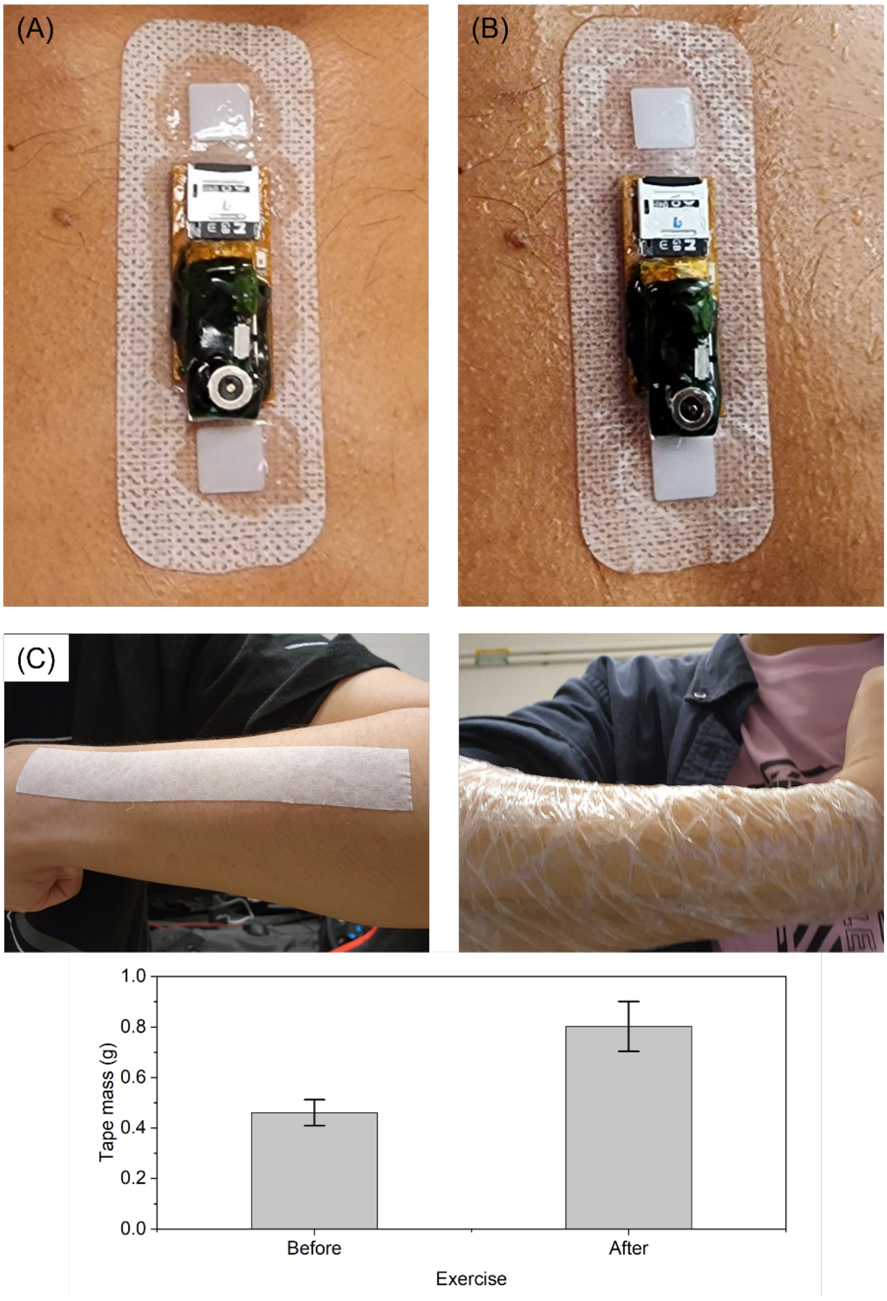


**Figure S2. Before and after status of sweaty skin region from jogging.** (A) Before running. (B) After running. (C) Tape soaking condition for adhesion force test.

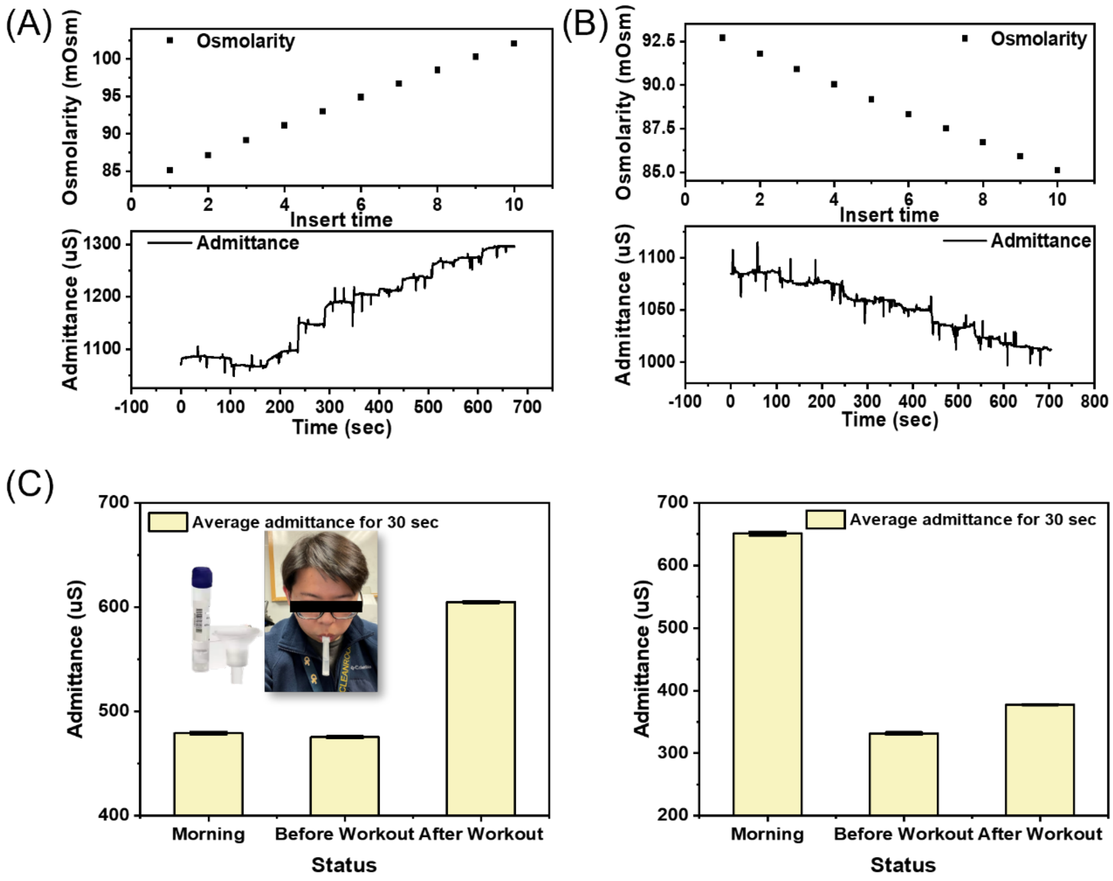


**Figure S3. Measured saliva admittance test results.** (A) Test result of continuous high stock osmotic solution addition, (B) Test result of continuous deionized water addition.

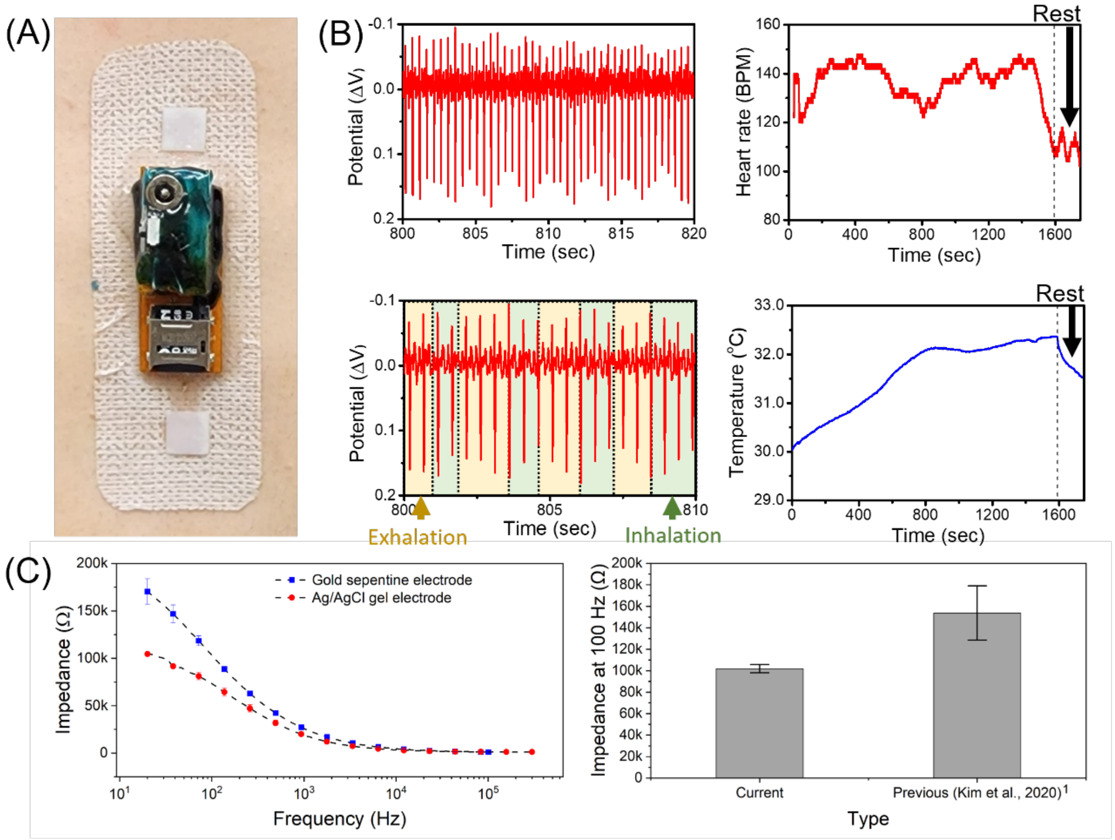


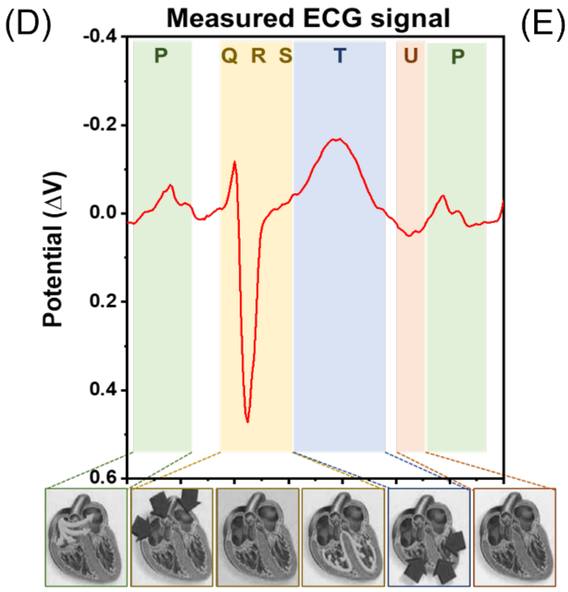


(E)

| | SNR (dB) | Position |
|---|---|---|
| This work | 32.55 | Chest |
| Y.S. Kim et.al (2022)[2] | 20.4 | Chest |
| L.W. Lo et.al (2022)[3] | 22.6 | Arm |
| M. Zulqarnain et.al (2020)[4] | 23.5 | Chest |
| A. Villegas et.al (2019)[5] | 21.7 | Arm |
| M. A. Zahed et al. (2023)[6] | 28 | Chest |
| M. Sharifuzzaman et al. (2023)[7] | 27 | Chest |

**Figure S4. Recorded electrocardiography data and calculated HR derivatives with signal analysis from an indoor treadmill test.** (A) Photo of chest patch attached to upper sternum. (B) Recorded electrocardiogram and its derivatives: HR (upper right), respiration (lower left), and temperature data. (C) Skin impedance from gold serpentine electrode on medical tape. (D) Analysis of electrocardiogram features including pqrstp peaks. (E) Comparative chart of signal-to-noise ratio relative to previous patch-type electrocardiogram devices.

**Table S1.** Data of a calibration curve using calibration standard samples on commercial saliva analyzer, gold-standard osmometer, and smart mouthguard

| Calibration study data set | | | | | | |
|---|---|---|---|---|---|---|
| **Osmolar calibration standard (mOsm)** | **MX3 Diagnostics osmometer** | | **Precision system osmo-mette** | | **Smart mouthguard** | |
| | Mean (mOsm) | SD | Mean (mOsm) | SD | Mean (mOsm) | SD |
| 0 | - | - | 3 | 0.70711 | - | - |
| 29 | - | - | 34 | 1.81659 | 23.39786 | 1.09958 |
| 58 | 47 | 5.654 | 62 | 0.70711 | 64.51507 | 1.43474 |
| 87 | 71 | 9.1869 | 93.5 | 2.68328 | 89.45088 | 1.39617 |
| 116 | 89 | 2.6842 | 125 | 1 | 121.8001 | 2.02687 |
| 145 | 125 | 3.5643 | 153 | 0.54772 | 155.446 | 2.62522 |
| 174 | 153 | 4.6845 | 182 | 0.83666 | 168.6336 | 2.43242 |
| 203 | 176 | 9.82341 | - | - | 191.0161 | 3.13063 |
| 232 | 205 | 5.6789 | - | - | 227.2842 | 2.83541 |

**Table S2.** Saliva sample test from human subjects in an environmental chamber

| Subject | Sampling | Body mass loss | Osmolality (mOsm) | | | Accuracy |
|---|---|---|---|---|---|---|
| | | | Gold standard osmometer | MX3 | Smart mouthguard | |
| Treadmill | Pre- | - | 86 | 74 | 97 | 113 % |
| | Post 1 | 1.54 % | 186 | 166 | 182 | 97.8 % |
| | Post 2 | 2.82 % | 221 | 185 | 207 | 91.1 % |
| Cycling | Pre | - | 82 | 72 | 80 | 97.5 % |
| | Post | 0.23 % | 91 | 65 | 88 | 96.7 % |

*Participant's average age: 25.6 years, average body mass:85.3 Kg, average resting HR: 82.3 bpm (n=3).

**Video S1.** Device test demonstration during mild activity.

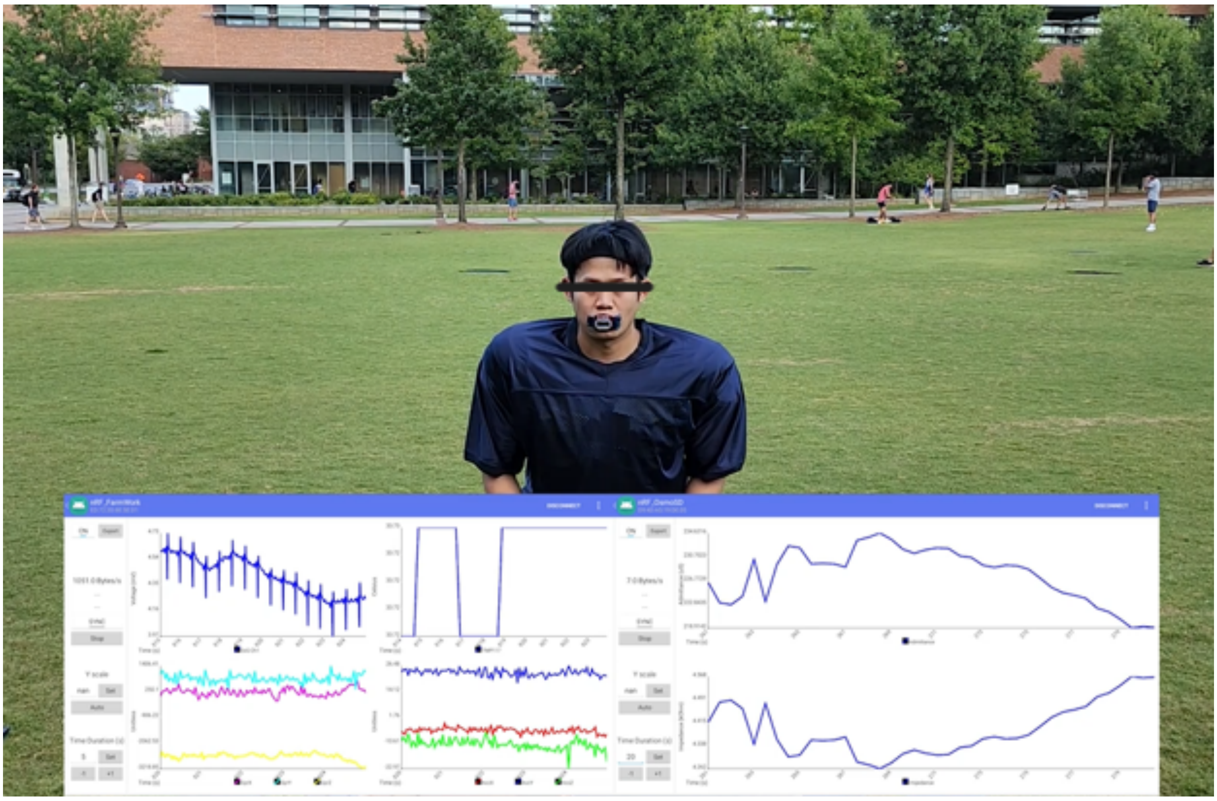

**Video S2.** Device test demonstration during treadmill running in an environmental chamber.

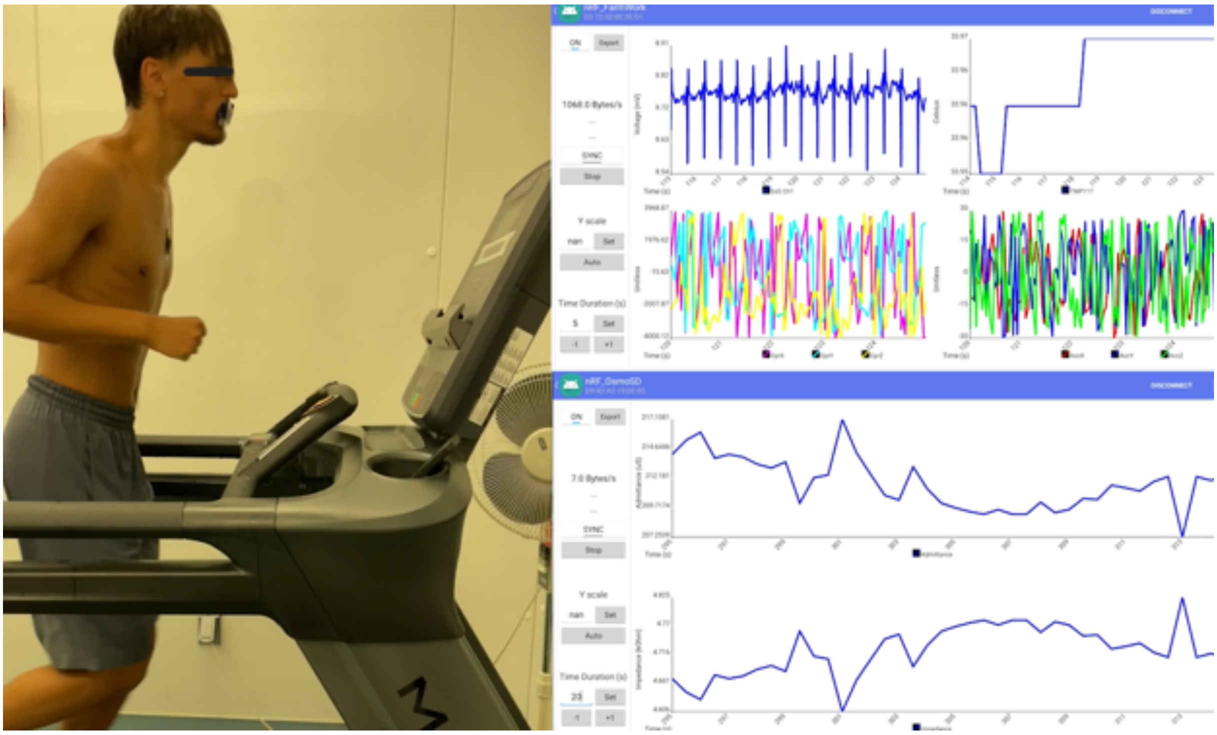

**References**

(1) H. Kim, Y.-S. Kim, M. Mahmood, S. Kwon, N. Zavanelli, H. S. Kim, Y. S. Rim, F. Epps, W.-H. Yeo, Advanced Science 2020, 7, 2000810.

(2) Y. S. Kim, J. Kim, R. Chicas, N. Xiuhtecutli, J. Matthews, N. Zavanelli, S. Kwon, S. H. Lee, V. S. Hertzberg, W. H. Yeo, *Adv Healthc Mater* **2022**, 11, e2200170.

(3) L.-W. Lo, J. Zhao, K. Aono, W. Li, Z. Wen, S. Pizzella, Y. Wang, S. Chakrabartty, C. Wang, *ACS Nano* **2022**, 16, 11792.

(4) M. Zulqarnain, S. Stanzione, G. Rathinavel, S. Smout, M. Willegems, K. Myny, E. Cantatore, *npj Flexible Electronics* **2020**, 4, 13.

(5) A. Villegas, D. McEneaney, O. Escalona, *Electronics* **2019**, 8, 1300.

(6) M. Sharifuzzaman, M. A. Zahed, M. S. Reza, M. Asaduzzaman, S. Jeong, H. Song, D. K. Kim, S. Zhang, J. Y. Park, Advanced Functional Materials 2023, 33, 2208894.

(7) M. A. Zahed, D. K. Kim, S. H. Jeong, M. Selim Reza, M. Sharifuzzaman, G. B. Pradhan, H. Song, M. Asaduzzaman, J. Y. Park, ACS Sensors 2023, 8, 2960.
